# DBU-promoted carboxylative cyclization of *o*-hydroxy- and *o*-acetamidoacetophenone

**DOI:** 10.3762/bjoc.11.102

**Published:** 2015-05-29

**Authors:** Wen-Zhen Zhang, Si Liu, Xiao-Bing Lu

**Affiliations:** 1 State Key Laboratory of Fine Chemicals, Dalian University of Technology, Dalian, 116024, P. R. China

**Keywords:** acyl migration, carbon dioxide, carboxylation, cyclization, condensation

## Abstract

The carboxylative cyclization of *o*-hydroxy- and *o*-acetamidoacetophenone with carbon dioxide promoted by the organic base 1,8-diazabicycloundec-7-ene (DBU) is reported. This reaction provides convenient access to the biologically important compounds 4-hydroxy-2*H*-chromen-2-one and 4-hydroxy-2(1*H*)-quinolinone in moderate to good yields using carbon dioxide as the carboxylation reagent. An acyl migration from nitrogen to carbon is observed in the reaction of *o*-acetamidoacetophenone.

## Introduction

**Figure 1:**
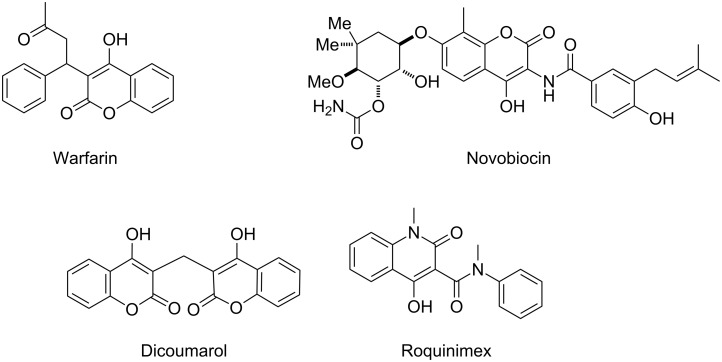
Selected examples for biologically active 4-hydroxy-2*H*-chromen-2-one and 4-hydroxy-2(1*H*)-quinolinone compounds.

4-Hydroxy-2*H*-chromen-2-ones and 4-hydroxy-2(1*H*)-quinolinones are key structural subunits found in many natural products [1], commercial drugs [2–3] and pharmacologically potent compounds (Figure 1) [4–5]. Warfarin, for example, is an anticoagulant widely used to prevent thrombosis [2]; Novobiocin has long been established as an aminocoumarin antibiotic [3]. Recent studies revealed that the anticoagulant Dicumarol is able to inhibit the growth of pancreatic cancer [4]. Roquinimex was reported as an antineoplastic agent [5]. Traditional methods for accessing these compounds rely heavily on cyclization reactions using diethyl carbonate in the presence of inorganic bases [6–7] or Friedel–Crafts reactions using strong and corrosive acids [8]. In terms of availability and toxicity of the starting materials, environmental benignity and economical concerns, the development of an alternative method for the synthesis of these compounds using carbon dioxide as the carboxylation reagent [9–16] is highly desirable.

It was previously reported that the α C–H bond in aromatic ketones readily undergoes a carboxylation reaction with carbon dioxide in the presence of a suitable base, producing β-ketocarboxylic acids [17–20]. Given that *o*-hydroxy- or *o*-acetamidoacetophenone is used as the starting material to react with carbon dioxide, the intramolecular carboxylative cyclization might provide a convenient access to 4-hydroxy-2*H*-chromen-2-one and 4-hydroxy-2(1*H*)-quinolinone. Indeed, Da Re and Sandri reported in 1960 that *o*-hydroxyacetophenone derivatives react with carbon dioxide (4 MPa) in the presence of 3 equivalents of potassium carbonate at 130–170 °C, yielding 4-hydroxy-2*H*-chromen-2-ones in moderate yields [21]. From the viewpoints of solubility, efficiency, and ease of recovery and reuse, the use of an organic base rather than potassium carbonate in this reaction would be more promising. DBU and MTBD were previously reported as suitable bases to promote the carboxylation of α-C–H bonds in aromatic ketones with carbon dioxide [17–20]. In extension of our continuous efforts in developing catalytic transformations of carbon dioxide into value-added fine chemicals [20,22–23], we report herein the DBU-promoted carboxylative cyclization of *o*-hydroxy- and *o*-acetamidoacetophenones with carbon dioxide to give 4-hydroxy-2*H*-chromen-2-ones and 4-hydroxy-2(1*H*)-quinolinones, respectively, in moderate to good yields under mild reaction conditions. An acyl migration from the nitrogen to carbon is observed in the reaction of *o*-acetamidoacetophenone.

## Results and Discussion

We started our investigation with the carboxylative cyclization of *o*-hydroxypropiophenone (**1a**) with carbon dioxide to identify the optimal organic base and reaction conditions (Table 1). The use of potassium carbonate as base in DMF at 100 °C gave 29% yield of product **2a** (Table 1, entry 1). When DBU and MTBD were used in this reaction instead of potassium carbonate, a significantly increased yield of **2a** was obtained (Table 1, entries 2 and 3). When switching the solvent to DMSO, further increased yields were obtained, whereby DBU showed a higher efficiency than MTBD (Table 1, entries 4 and 5). Other solvents such as DMAc and THF gave dramatically decreased yields (Table 1, entries 6 and 7). Unexpectedly, we found that a decrease of temperature from 100 °C to 80 °C in DMSO led to a higher yield (87%) of **2a** (Table 1, entry 8). The reaction was found to be sensitive to the carbon dioxide pressure and performing the reaction at a lower pressure gave a distinctly decreased yield (Table 1, entry 10). When the reaction was conducted under atmospheric carbon dioxide, no carboxylative cyclization product was obtained (Table 1, entry 11). Therefore, the optimal reaction conditions were established as following: 2.0 equiv DBU as base, 3.0 MPa of carbon dioxide, DMSO as solvent at 80 °C for 24 h.

**Table 1:** Optimization of the reaction conditions.^a^

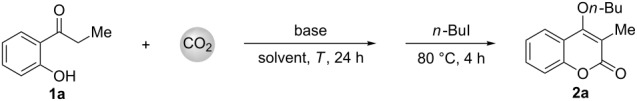					

Entry	Base	Solvent	*T*/°C	*p*(CO_2_)/MPa	Yield/%^b^

1	K_2_CO_3_	DMF	100	3	29
2	DBU	DMF	100	3	49
3	MTBD	DMF	100	3	65
4	MTBD	DMSO	100	3	68
5	DBU	DMSO	100	3	75
6	DBU	DMAc	100	3	32
7	DBU	THF	100	3	10
**8**	**DBU**	**DMSO**	**80**	**3**	**87**
9	DBU	DMSO	60	3	65
10	DBU	DMSO	80	2	53
11	DBU	DMSO	80	0.1	<1

^a^Reaction conditions: *o*-hydroxyacetophenone (**1a**, 0.5 mmol), base (1 mmol), solvent (2 mL), 24 h; then *n-*BuI (1.0 mmol), 80 °C, 4 h. ^b^Isolated yield.

Under the optimal reaction conditions, the substrate scope was then investigated (Table 2). Compared with *o*-hydroxypropiophenone, *o*-hydroxyacetophenone gave a slightly lower yield of the 2*H*-chromen-2-one product (Table 2, entries 2 and 4). *o*-Hydroxyacetophenone bearing electron-donating alkyl and ether groups, or electron-withdrawing fluoro and bromo groups undergoes the carboxylative cyclization reaction smoothly, affording the corresponding 4-butoxy-2*H*-chromen-2-ones **2b**–**2f** in moderate to good yields (Table 2, entries 2–6). The bromo group in product **2f** and the alkyne group in product **2g** offer opportunities for further functionalization of these 2*H*-chromen-2-ones using well-established methods [24] (Table 2, entries 6 and 7). 2-Hydroxy-1-acetylnaphthalene (**1h**) participates in the carboxylative cyclization reaction to furnish the tricyclic product **2h** in moderate yield (Table 2, entry 8).

**Table 2:** Carboxylative cyclization of various *o*-hydroxyacetophenones with carbon dioxide.^a^

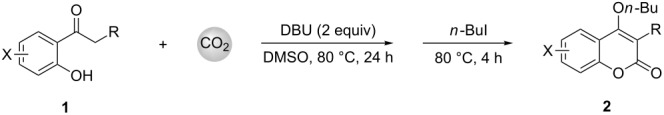			

Enty	Substrate	Product	Yield/%^b^

1	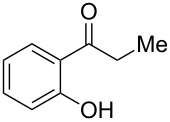 **1a**	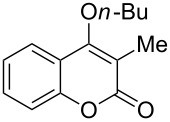 **2a**	87
2	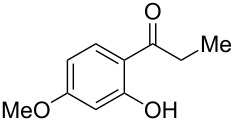 **1b**	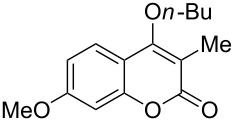 **2b**	79
3	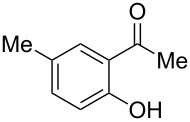 **1c**	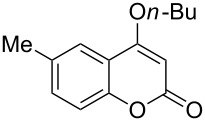 **2c**	56
4	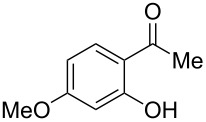 **1d**	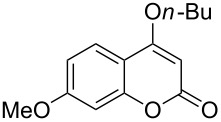 **2d**	45
5	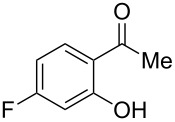 **1e**	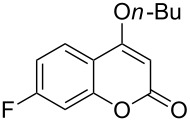 **2e**	49
6	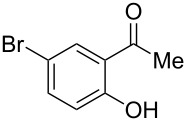 **1f**	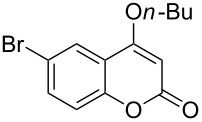 **2f**	36
7	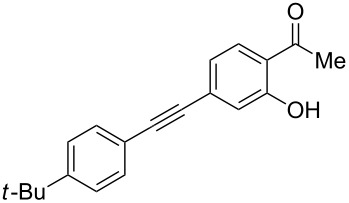 **1g**	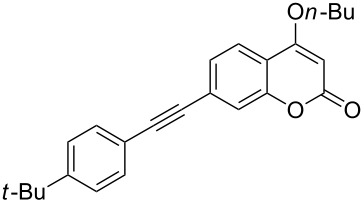 **2g**	65
8	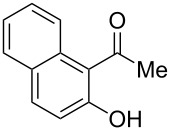 **1h**	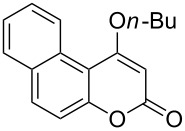 **2h**	42

^a^Reaction conditions: *o*-hydroxyacetophenone (**1**) (0.5 mmol), DBU (1.0 mmol), CO_2_ (3.0 MPa), DMSO (2 mL), 80 °C, 24 h; then *n*-BuI (1.0 mmol), 80 °C, 4 h. ^b^Isolated yield.

With the successful DBU-promoted carboxylative cyclization of *o*-hydroxyacetophenone at hand, we then extended this strategy to *o*-acetamidoacetophenone to synthesize 4-hydroxy-2(1*H*)-quinolinone (Table 3). Using 4 equivalents DBU as base in DMSO at 80 °C, *o*-acetamidoacetophenone (**3a**) underwent the carboxylative cyclization reaction to provide 3-acetyl-4-methoxy-2(1*H*)-quinolinones **4a** and **5a** (Table 3, entry 1). Noteworthy, the acyl group was no longer bound to nitrogen in the product, which implies that a nitrogen to carbon acyl migration occurred during the reaction. The derivatization reaction using iodide compounds at higher temperature led to complex product mixtures. *o*-Acetamidoacetophenone substrates containing methoxy (**3b**) and bromo (**3c**) groups also reacted smoothly to afford the corresponding products (Table 3, entries 2 and 3). The reactions using benzamido- (**3d**) and *p*-toluenesulfonamido- (**3e**) acetophenone gave complex mixtures and no carboxylative cyclization product was observed (Table 3, entries 4 and 5).

**Table 3:** Carboxylative cyclization of various *o*-acetamidoacetophenones with carbon dioxide.^a^

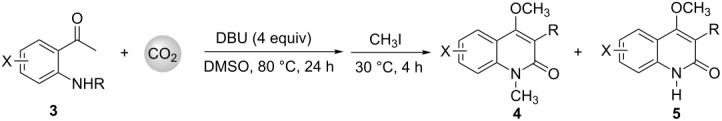				

Entry	Substrate	Product		Yield/%
		**4**	**5**	**4** + **5**

1	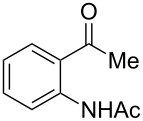 **3a**	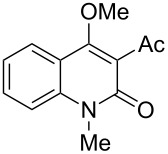 **4a**	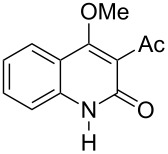 **5a**	42 + 35
2	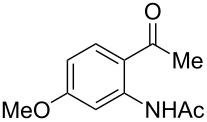 **3b**	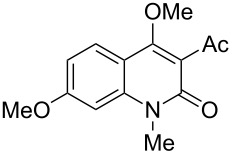 **4b**	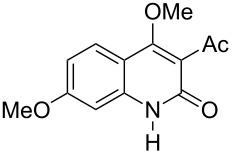 **5b**	38 + 37
3	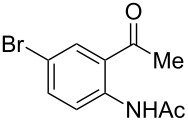 **3c**	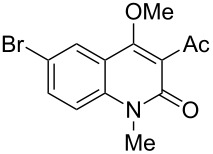 **4c**	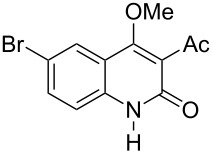 **5c**	32 + 20
4	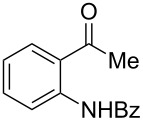 **3d**	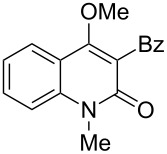 **4d**	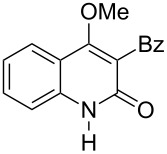 **5d**	<1
5	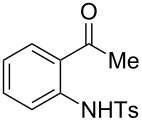 **3e**	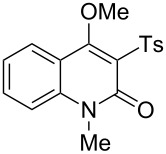 **4e**	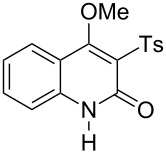 **5e**	<1

^a^Reaction conditions: *o*-acetamidoacetophenone (**3**, 0.5 mmol), DBU (2.0 mmol), CO_2_ (3.0 MPa), DMSO (2 mL), 80 °C, 24 h; then MeI (2.0 mmol), 30 °C, 4 h. ^b^Isolated yield of separated products.

**Scheme 1:**
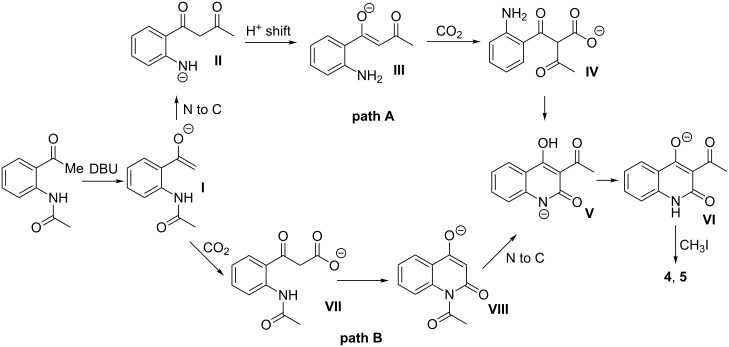
Possible mechanism for the carboxylative cyclization of *o*-acetamidoacetophenone.

A likely mechanism for the carboxylative cyclization of *o*-acetamidoacetophenone with carbon dioxide is proposed as shown in Scheme 1. The reaction can evolve along two pathways: in path **A**, deprotonation of *o*-acetamidoacetophenone by DBU gives enolate **I**, which undergoes an acyl migration from nitrogen to carbon [25–26] similar to the Baker–Venkataraman O to C acyl migration [27]. After a proton shift from the enol to nitrogen, the resultant intermediate **III** is carboxylated with carbon dioxide in the presence of DBU to afford intermediate **IV**, which subsequently undergoes a cyclization reaction to give **V**. The product is obtained after derivatization with methyl iodide. Also, path **B** in which the N to C acyl migration occurs after the carboxylative cyclization cannot be excluded.

**Scheme 2:**
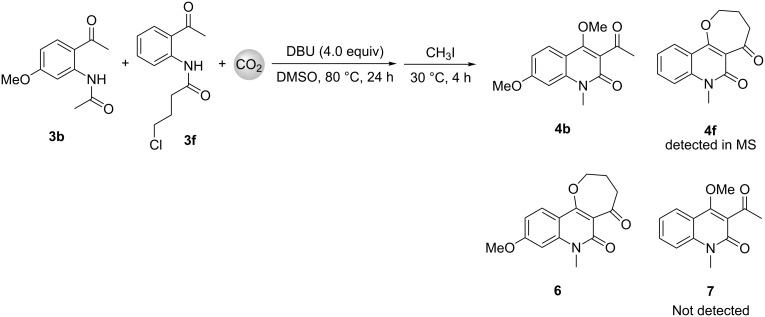
Cross carboxylative cyclization reaction.

We also conducted a cross experiment as shown in Scheme 2. When compounds **3b** and **3f** were reacted concomitantly, the corresponding carboxylative cyclization products **4b** and **4f** were obtained. No cross products **6** and **7** were detected, which implies that the N to C acyl shift occurred intramolecularly, not intermolecularly.

## Conclusion

In summary, we have developed a DBU-promoted carboxylative cyclization of *o*-hydroxy- and *o*-acetamidoacetophenones with carbon dioxide. This methodology provides a convenient access to the biologically important 4-hydroxy-2*H*-chromen-2-ones and 4-hydroxy-2(1*H*)-quinolinones in moderate to good yields under mild reaction conditions. While there are precedents for the carboxylation of enolates, a practical protocol was developed that relies on in situ cyclization to form thermodynamically stable coumarins. Importantly, the use of an intramolecular in situ trap avoids the problem of decarboxylation during workup. In case of *o*-acetamidoacetophenones, an acyl migration from nitrogen to carbon was observed. The cross experiment showed that the N to C acyl shift occurred intramolecularly.

## Experimental

Similarly as described in our previous paper [22], a 20 mL oven-dried autoclave containing a stirring bar was charged with *o*-hydroxyacetophenone (**1**) or *o*-acetamidoacetophenone (**3**) (0.5 mmol), DBU (1.0 mmol for **1**, 2.0 mmol for **3**), and 2 mL dry DMSO. After purging the autoclave with CO_2_ three times, the sealed autoclave was pressurized to the appropriate pressure with CO_2_. The reaction mixture was stirred at 80 °C for 24 h, then the autoclave was cooled to room temperature and the remaining CO_2_ was vented slowly. Then *n-*BuI (1.0 mmol for **1**) or MeI (2.0 mmol for **3**) was added into the autoclave and the reaction mixture was stirred at 80 °C (for **1**) or at 30 °C (for **3**) for 4 h. The reaction mixture was then diluted with water (30 mL) and extracted with ethyl acetate (3 × 30 mL). The combined organic layers were washed with water and brine, dried over Na_2_SO_4_ and filtered. The solvent was removed under vacuum. The product was isolated by column chromatography on silica gel (hexane/ethyl acetate 2:1).

## Supporting Information

File 1 Experimental procedures, spectroscopic and analytical data, and copies of NMR spectra of the products.
