# Supplementary material for: DBU-promoted carboxylative cyclization of o-hydroxy- and o-acetamidoacetophenone
Source: Beilstein J Org Chem. 2015 May 29;11:906–12. doi: 10.3762/bjoc.11.102 (PMC4464362; doi:10.3762/bjoc.11.102)
Supplement: File 1 — Experimental procedures, spectroscopic and analytical data, and copies of NMR spectra of the products. [file Beilstein_J_Org_Chem-11-906-s001.pdf]

Supporting Information
for
DBU-promoted carboxylative cyclization of *o*-hydroxy- and *o*-acetamidoacetophenone

Wen-Zhen Zhang*, Si Liu and Xiao-Bing Lu

Address: State Key Laboratory of Fine Chemicals, Dalian University of Technology, Dalian, 116024, P. R. China

Email: Wen-Zhen Zhang - zhangwz@dlut.edu.cn;

* Corresponding author

Experimental procedures, spectroscopic and analytical data, and copies of NMR spectra of the products

Table of contents

1. General methods S2

2. General procedure for reaction of *o*-hydroxyacetophenone with CO_2..............................S2

3. General procedure for reaction of *o*-acetamidoacetophenone with CO_2...........................S6

4. ^1H NMR and ^{13}C NMR spectra of the products S8

1. General methods

Unless otherwise stated, all manipulations were performed using standard Schlenk techniques under a dry nitrogen or carbon dioxide atmosphere. DMF, DMAc and DMSO were distilled from CaH_2 at 60 °C under reduced pressure and stored over 4 Å molecular sieves. Column chromatography was performed on silica gel (200–300 mesh). Thin layer chromatography was performed on 0.20 mm GF254 plates. Visualization was accomplished with UV light (254 nm), cerium ammonium molybdate, or potassium permanganate.

NMR spectra were recorded on a Bruker Avance II 400M type (^{1}H NMR, 400 MHz; ^{13}C NMR, 100 MHz) spectrometer in $CDCl_3$ at ambient temperature and chemical shifts are expressed in parts per million (δ, ppm). Proton chemical shifts are referenced to 7.26 ppm ($CHCl_3$) and carbon chemical shifts are referenced to 77.0 ppm ($CDCl_3$). Data reporting uses the following abbreviations: s, singlet; d, doublet; t, triplet; m, multiplet; hept, heptet, and *J*, coupling constant in Hz. High resolution mass spectra (HRMS) were recorded on a Q-TOF mass spectrometry (Micromass, Wythenshawe, UK) equipped with Z-spray ionization source. Infrared spectra (IR) were measured using a Nicolet NEXUS FTIR spectrophotometer.

Unless otherwise indicated, commercially available starting materials were purchased from Energy Chemical.

2. General procedure for reaction of *o*-hydroxyacetophenone with CO_2

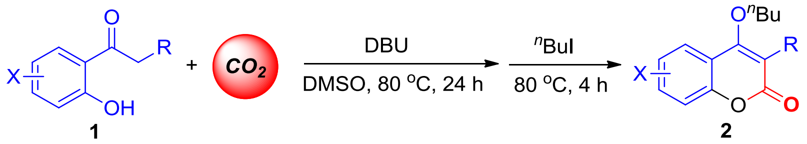

Similarly as described in our previous paper (*Catal. Sci. Technol.* **2014**, *4*, 1570), a 20 mL oven-dried autoclave containing a stirring bar was charged with *o*-hydroxyacetophenone (**1**) (0.5 mmol), DBU (1.0 mmol), and 2 mL dry DMSO. After purging the autoclave with CO_2 three times, the sealed autoclave was pressurized to the appropriate pressure with CO_2. The reaction mixture was stirred at 80 °C for 24 h, then the autoclave was cooled to room temperature and the remaining CO_2 was vented slowly. Then *n*-BuI (1.0 mmol) was added into the autoclave and the reaction mixture was stirred at 80 °C for 4 h. The reaction mixture was then diluted with water (30 mL) and extracted with ethyl acetate (3 × 30 mL). The combined organic layers were washed with water and brine, dried over Na_2SO_4 and filtered. The solvent was removed under vacuum. The product was isolated by column chromatography on silica gel (hexane/ethyl acetate 2:1).

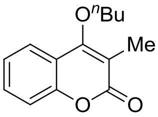 **4-Butoxy-3-methyl-2*H*-chromen-2-one (2a)**. 87% yield. **^{1}H NMR** (400 MHz, $CDCl_3$) δ 7.70 (dd, *J* = 7.9, 1.5 Hz, 1H), 7.52-7.47 (m, 1H), 7.34-7.28 (m, 2H), 4.12 (t, *J* = 6.6 Hz, 2H), 2.17 (s, 3H), 1.90-1.83 (m, 2H), 1.61-1.52 (m, 2H), 1.02 (t, *J* = 7.4 Hz, 3H). **^{13}C NMR** (100 MHz, $CDCl_3$) δ 164.43, 163.01, 152.29, 131.13, 124.00 , 123.02, 117.74, 116.63, 112.39, 74.12, 32.36, 19.17, 13.86, 10.94. **IR** (neat, cm^{-1}) ν 2961, 2876, 1704, 1627, 1606, 1454, 1343, 1323, 1287, 1216, 1073, 1008, 959, 784, 760, 742. **HRMS** (ESI, *m*/*z*) calcd for $C_{14}H_{17}O_3$ $[M+H]^+$: 233.1178, found: 233.1169.

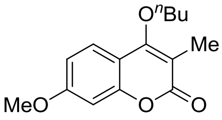 **4-Butoxy-3-methyl-7-methoxy-2*H*-chromen-2-one (2b)**. 79% yield. **^{1}H NMR** (400 MHz, $CDCl_3$) δ 7.59 (d, *J* = 8.7 Hz, 1H), 6.87-6.82 (m, 2H), 4.10 (t, *J* = 6.5 Hz, 2H), 3.87 (s, 3H), 2.13 (s, 3H), 1.87-1.81 (m, 2H), 1.58-1.52 (m, 2H), 1.01 (t, *J* = 7.4 Hz, 3H). **^{13}C NMR** (100 MHz, $CDCl_3$) δ 164.86, 163.49, 162.23, 153.95, 124.05, 112.14, 111.10, 109.13, 100.48, 74.03, 55.70, 32.36, 19.15, 13.83, 10.69. **IR** (neat, cm^{-1}) ν 2958, 2928, 2873, 1711, 1614, 1508, 1443, 1378, 1357, 1336, 1248, 1160, 1104, 1064, 1028, 969, 835, 758, 737. **HRMS** (EI, *m*/*z*) calcd for $C_{15}H_{18}O_4$ $[M]^+$: 262.1205, found: 262.1208.

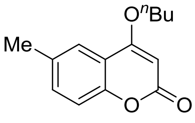 **4-Butoxy-6-methyl-2*H*-chromen-2-one (2c)**. 56% yield. **^{1}H NMR** (400 MHz, $CDCl_3$) δ 7.59 (s, 1H), 7.35 (d, *J* = 8.4 Hz, 1H), 7.21 (d, *J* = 8.4 Hz, 1H), 5.65 (s, 1H), 4.13 (t, *J* = 6.4 Hz, 2H), 2.42 (s, 3H), 1.93-1.88 (m, 2H), 1.61-1.53 (m, 2H), 1.03 (t, *J* = 7.4 Hz, 3H). **^{13}C NMR** (100 MHz, $CDCl_3$) δ 165.79, 163.32, 151.54, 133.55, 133.32, 122.65, 116.56, 115.50, 90.33, 69.08, 30.53, 20.93, 19.23, 13.75. **IR** (neat, cm^{-1}) ν 3077, 2953, 1732, 1633, 1608, 1577, 1444, 1370, 1209, 1189, 1106, 935, 856, 827. **HRMS** (ESI, *m*/*z*) calcd for $C_{14}H_{17}O_3$ $[M+H]^+$: 233.1178, found: 233.1172.

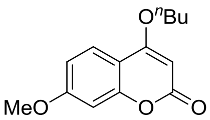 **4-Butoxy-7-methoxy-2*H*-chromen-2-one (2d)**. 45% yield. **^{1}H NMR** (400 MHz, $CDCl_3$) δ 7.58 (d, *J* = 8.8 Hz, 1H), 6.86-6.80 (m, 2H), 4.09 (t, *J* = 6.6 Hz, 2H),

3.86 (s, 3H), 2.13 (s, 3H), 1.88-1.81 (m, 2H), 1.60-1.50 (m, 2H), 1.01 (t, *J* = 7.4 Hz, 3H). **^{13}C NMR** (100 MHz, $CDCl_3$) δ 166.39, 163.78, 163.39, 155.39, 124.31, 112.36, 109.32, 100.63, 88.12, 69.21, 55.96, 30.77, 19.45, 13.99. **IR** (neat, cm^{-1}) ν 3055, 2957, 2927, 1707, 1620, 1456, 1422, 1381, 1153, 1030, 970, 908, 882. **HRMS** (ESI, *m*/*z*) calcd for $C_{14}H_{17}O_4$ $[M+H]^+$: 249.1127, found: 249.1128.

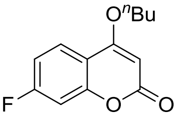

4-Butoxy-7-fluoro-2*H*-chromen-2-one (2e). 49% yield. **^{1}H NMR** (400 MHz, $CDCl_3$) δ 7.82 (dd, *J* = 8.7, 6.1 Hz, 1H), 7.02-6.98 (m, 2H), 5.63 (s, 1H), 4.14 (t, *J* = 6.4 Hz, 2H), 1.91-1.86 (m, 2H), 1.58-1.52 (m, 2H), 1.02 (t, *J* = 7.4 Hz, 3H). **^{13}C NMR** (100 MHz, $CDCl_3$) δ 165.38, 164.93, 162.74, 154.57, 124.92, 111.96, 104.19, 89.42, 69.27, 53.43, 30.47, 19.19, 13.72. **IR** (neat, cm^{-1}) ν 3079, 2962, 2934, 1734, 1632, 1508, 1457, 1422, 1382, 1262, 1140, 1113, 994, 965, 843, 818, 741. **HRMS** (ESI, *m*/*z*) calcd for $C_{13}H_{13}FO_3Na$ $[M+Na]^+$: 259.0746, found: 259.0741.

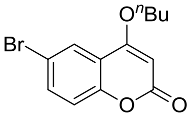

4-Butoxy-6-bromo-2*H*-chromen-2-one (2f). 36% yield. **^{1}H NMR** (400 MHz, $CDCl_3$) δ 7.92 (s, 1H), 7.63 (d, *J* = 8.8 Hz, 1H), 7.21 (d, *J* = 8.8 Hz, 1H), 5.69 (s, 1H), 4.14 (t, *J* = 6.4 Hz, 2H), 1.94-1.87 (m, 2H), 1.58-1.51 (m, 2H), 1.03 (t, *J* = 7.4 Hz, 3H). **^{13}C NMR** (100 MHz, $CDCl_3$) δ 164.52, 162.27, 152.21, 135.14, 125.68, 118.56, 117.43, 116.68, 91.07, 69.48, 30.43, 19.18, 13.71. **IR** (neat, cm^{-1}) ν 2965, 2920, 1736, 1624, 1602, 1467, 1430, 1359, 1265, 1243, 1191, 1112, 1022, 925, 826, 796, 749, 730, 702. **HRMS** (ESI, *m*/*z*) calcd for $C_{13}H_{14}BrO_3$ $[M+H]^+$: 297.0126, found: 297.0132.

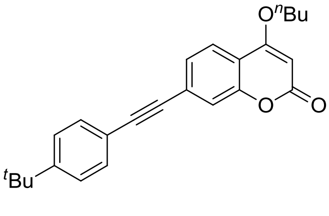

4-Butoxy-7-(4-*tert*-butylphenylethynyl)-2*H*-chromen-2-one (2g). 65% yield. **^{1}H NMR** (400 MHz, $CDCl_3$) δ 7.78 (d, *J* = 8.2 Hz, 1H), 7.50 (d, *J* = 8.4 Hz, 2H), 7.44-7.39 (m, 4H), 5.67 (s, 1H), 4.14 (t, *J* = 6.4 Hz, 2H), 1.92-1.86 (m, 2H), 1.59-1.53 (m, 2H), 1.34 (s, 10H), 1.03 (t, *J* = 7.4 Hz, 3H). **^{13}C NMR** (100 MHz, $CDCl_3$) δ 164.30, 161.77,

152.10, 151.36, 130.54, 126.76, 126.04, 124.47, 121.94, 118.35, 114.38, 91.97, 89.56, 86.47, 68.22, 33.87, 30.13, 29.48, 28.68, 18.18, 12.71. **IR** (neat, cm^{-1}) ν 2961, 2925, 2855, 2213, 1727, 1617, 1499, 1462, 1416, 1376, 1261, 1094, 1021, 870, 800. **HRMS** (ESI, *m*/*z*) calcd for $C_{25}H_{27}O_3$ $[M+H]^+$: 375.1960, found: 375.1955.

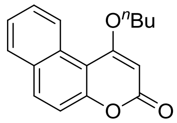

4-Butoxy-3*H*-benzo[*f*]chromen-3-one (2h). 42% yield. **^{1}H NMR** (400 MHz, $CDCl_3$) δ 9.10 (d, *J* = 8.7 Hz, 1H), 7.93 (d, *J* = 9.0 Hz, 1H), 7.85 (d, *J* = 8.0 Hz, 1H), 7.63-7.50 (m, 2H), 7.39 (d, *J* = 7.3 Hz, 1H), 5.78 (s, 1H), 4.21 (t, *J* = 6.4 Hz, 2H), 2.06-1.99 (m, 2H), 1.68-1.58 (m, 2H), 1.05 (t, *J* = 7.4 Hz, 3H). **^{13}C NMR** (100 MHz, $CDCl_3$) δ 169.64, 162.70, 154.66, 134.18, 130.89, 129.09, 128.34, 126.24, 125.67, 117.39, 109.10, 90.79, 69.96, 30.67, 19.50, 13.89. **IR** (neat, cm^{-1}) ν 2954, 2919, 2850, 1737, 1587, 1564, 1461, 1428, 1380, 1322, 1263, 1242, 1209, 1192, 1145, 1070, 940, 923, 815, 741. **HRMS** (ESI, *m*/*z*) calcd for $C_{17}H_{17}O_3$ $[M+H]^+$: 269.1178, found: 269.1179.

3. General procedure for reaction of *o*-acetamidoacetophenone with CO_2

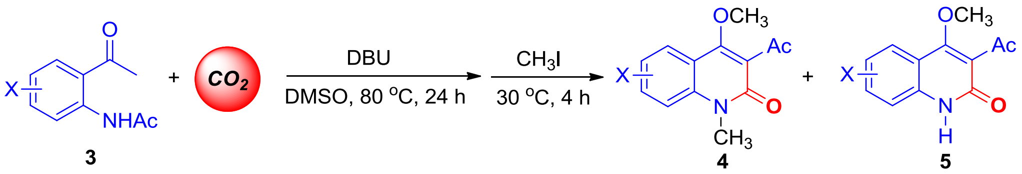

Similarly as described in our previous paper (*Catal. Sci. Technol.* **2014**, *4*, 1570), a 20 mL oven-dried autoclave containing a stirring bar was charged with *o*-acetamidoacetophenone (**3**, 0.5 mmol), DBU (2.0 mmol), and 2 mL dry DMSO. After purging the autoclave with CO_2 three times, the sealed autoclave was pressurized to the appropriate pressure with CO_2. The reaction mixture was stirred at 80 °C for 24 h, then the autoclave was cooled to room temperature and the remaining CO_2 was vented slowly. Then CH_3I (2.0 mmol) was added into the autoclave and the reaction mixture was stirred at 30 °C for 4 h. The reaction mixture was diluted with water (30 mL) and extracted with ethyl acetate (3 × 30 mL). The combined organic layers were washed with water and brine, dried over Na_2SO_4 and filtered. The solvent was removed under vacuum. The product was isolated by column chromatography on silica gel (hexane/ethyl acetate 2:1).

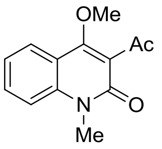

3-Acetyl-4-methoxy-1-methyl-2(1*H*)-quinolinone (4a). 42% yield. ^{1}**H NMR** (400 MHz, $CDCl_3$) δ 7.78 (dd, *J* = 8.1, 1.1 Hz, 1H), 7.64-7.60 (m, 1H), 7.38 (d, *J* = 8.5 Hz, 1H), 7.30 (d, *J* = 7.4 Hz, 1H), 3.97 (s, 3H), 3.72 (s, 3H), 2.47 (s, 3H). ^{13}**C NMR** (100 MHz, $CDCl_3$) δ 167.21, 159.11, 143.74, 139.45, 131.50, 126.49, 125.92, 122.46, 120.39, 114.53, 52.64, 29.46, 16.35. **IR** (neat, cm^{-1}) ν 2953, 2917, 2250, 1737, 1643, 1596, 1461, 1319, 1286, 1248, 1162, 1086, 910, 754, 729. **HRMS** (ESI, *m/z*) calcd for $C_{13}H_{14}NO_3$ $[M+H]^+$: 232.0974, found: 232.0974.

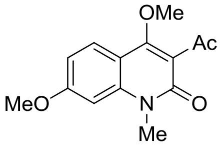

3-Acetyl-4,7-dimethoxy-1-methyl-2(1*H*)-quinolinone (4b). 38% yield. ^{1}**H NMR** (400 MHz, $CDCl_3$) δ 7.69 (d, *J* = 8.9 Hz, 1H), 6.87 (dd, *J* = 8.9, 2.3 Hz, 1H), 6.78 (d, *J* = 2.3 Hz, 1H), 3.96 (s, 3H), 3.93 (s, 3H), 3.68 (s, 3H), 2.43 (s, 3H). ^{13}**C NMR** (100 MHz, $CDCl_3$) δ 166.45, 161.31, 158.55, 142.93, 140.25, 126.47, 122.56, 113.37, 108.90, 97.77, 54.59, 51.51, 28.46, 15.29. **IR** (neat, cm^{-1}) ν 2957, 2905, 2245, 1736, 1639, 1598, 1445,

1306, 1224, 1079, 1049, 906, 828, 726. **HRMS** (ESI, *m*/*z*) calcd for $C_{14}H_{16}NO_4$ $[M+H]^+$: 262.1079, found: 262.1052.

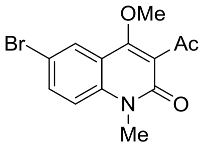

3-Acetyl-6-bromo-4-methoxy-1-methyl-2(1*H*)-quinolinone (4c). 32% yield. **^{1}H NMR** (400 MHz, $CDCl_3$) δ 7.87 (d, *J* = 1.9 Hz, 1H), 7.69 (dd, *J* = 9.0, 1.9 Hz, 1H), 7.25 (s, 1H), 3.97 (s, 3H), 3.69 (s, 3H), 2.43 (s, 3H). **^{13}C NMR** (100 MHz, $CDCl_3$) δ 165.67, 157.71, 141.56, 137.37, 133.16, 127.38, 126.46, 120.97, 115.20, 114.54, 51.71, 28.58, 15.29. **IR** (neat, cm^{-1}) ν 2950, 2927, 2252, 1729, 1647, 1587, 1557, 1493, 1243, 1168, 1108, 1052, 874, 807, 728. **HRMS** (ESI, *m*/*z*) calcd for $C_{13}H_{13}BrNO_3$ $[M+H]^+$: 310.0079, found: 310.0070.

4. ^{1}H NMR and ^{13}C NMR spectra of the products

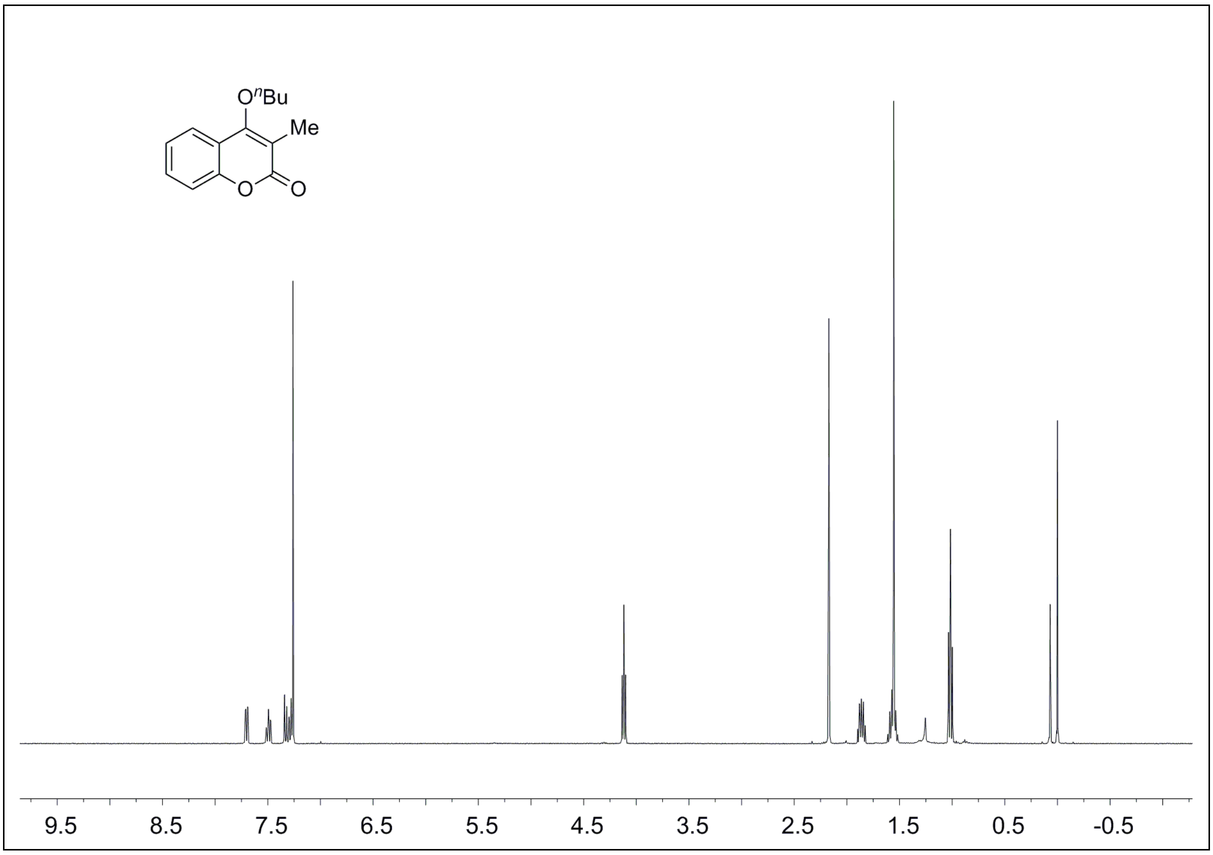

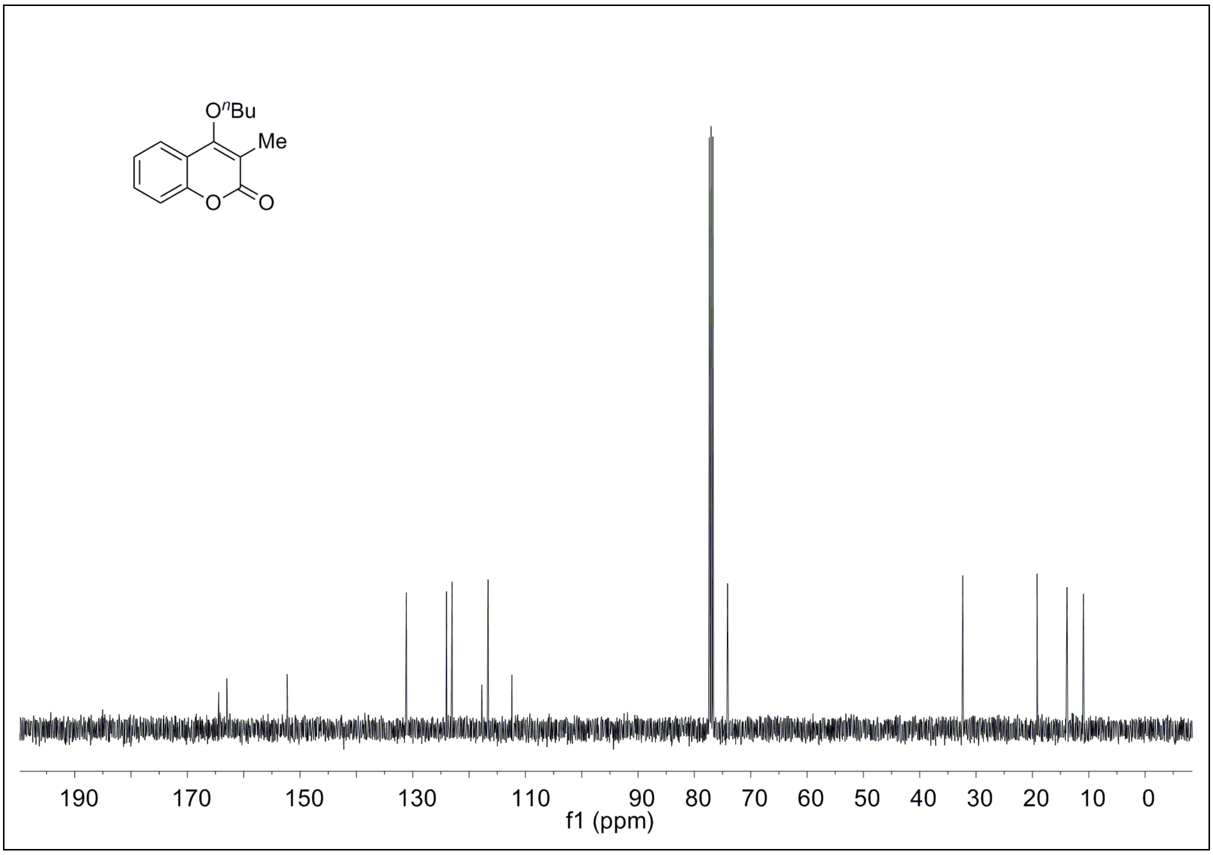

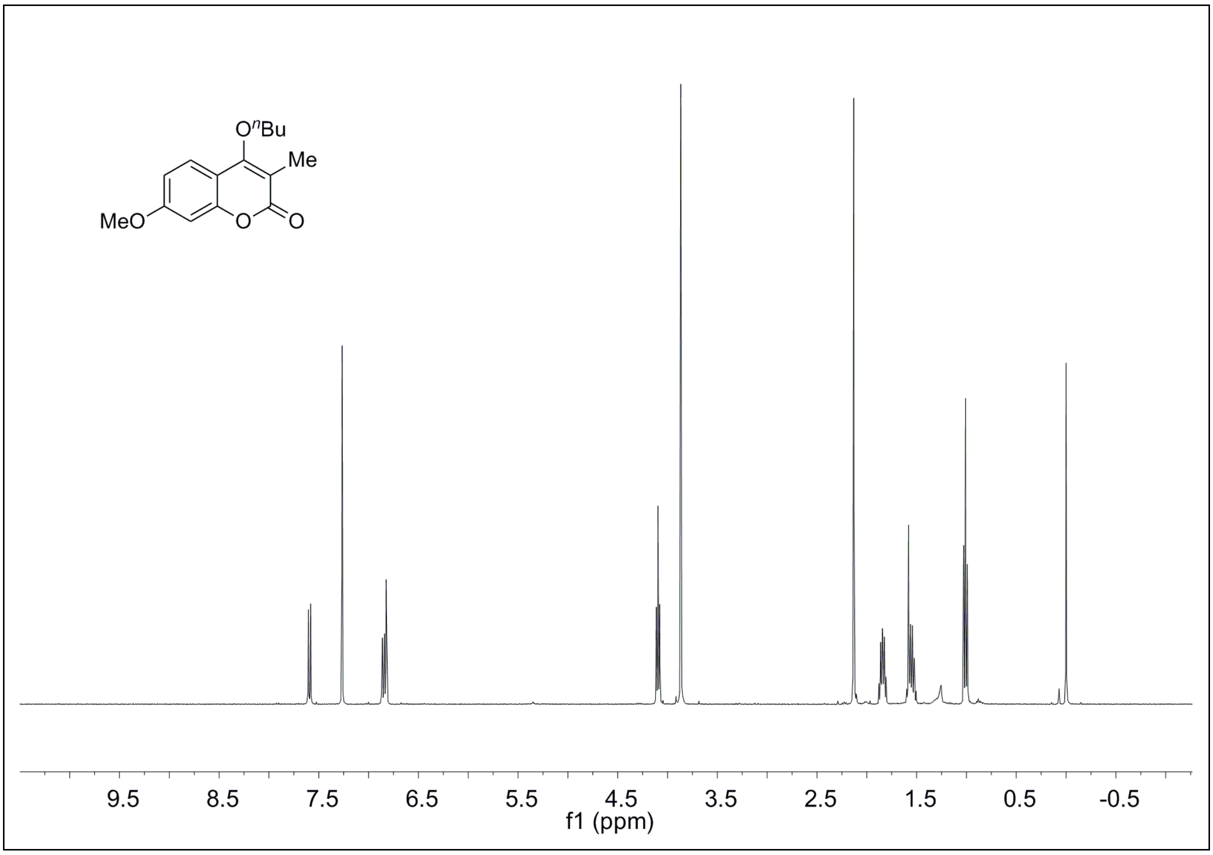
O^{n}Bu
Me
MeO
O
O
9.5
8.5
7.5
6.5
5.5
4.5
3.5
2.5
1.5
0.5
-0.5
f1 (ppm)

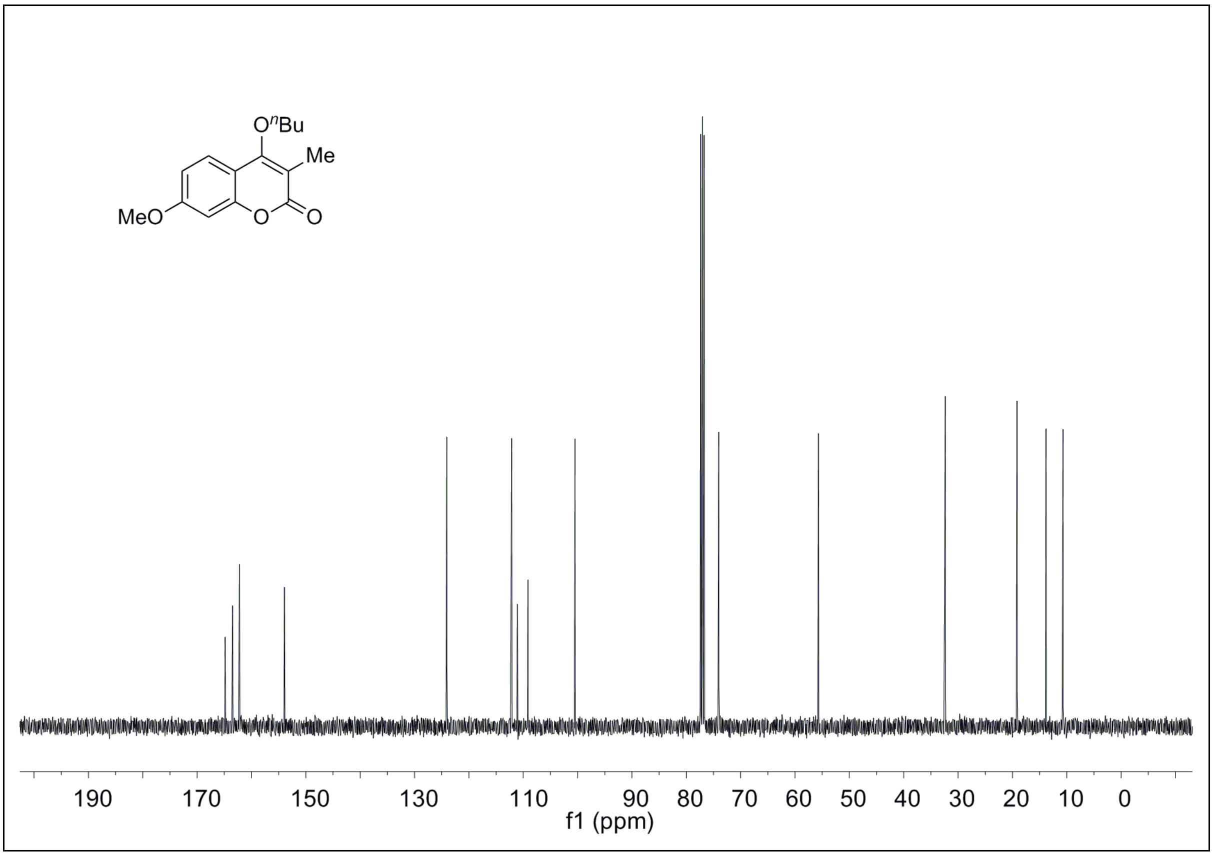
O^{n}Bu
Me
MeO
O
O
190
170
150
130
110
90
80
70
60
50
40
30
20
10
0
f1 (ppm)

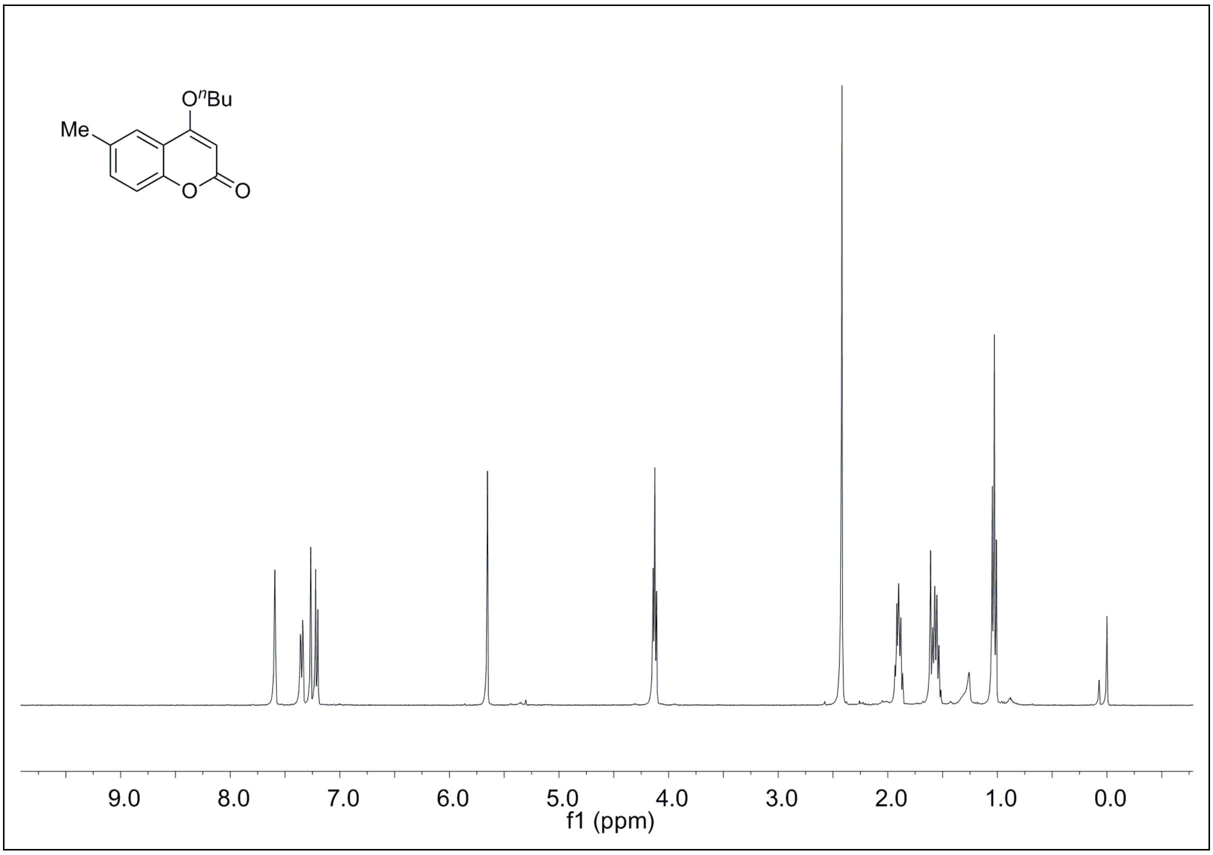
O^{n}Bu
Me
O
O
9.0
8.0
7.0
6.0
5.0
4.0
3.0
2.0
1.0
0.0
f1 (ppm)

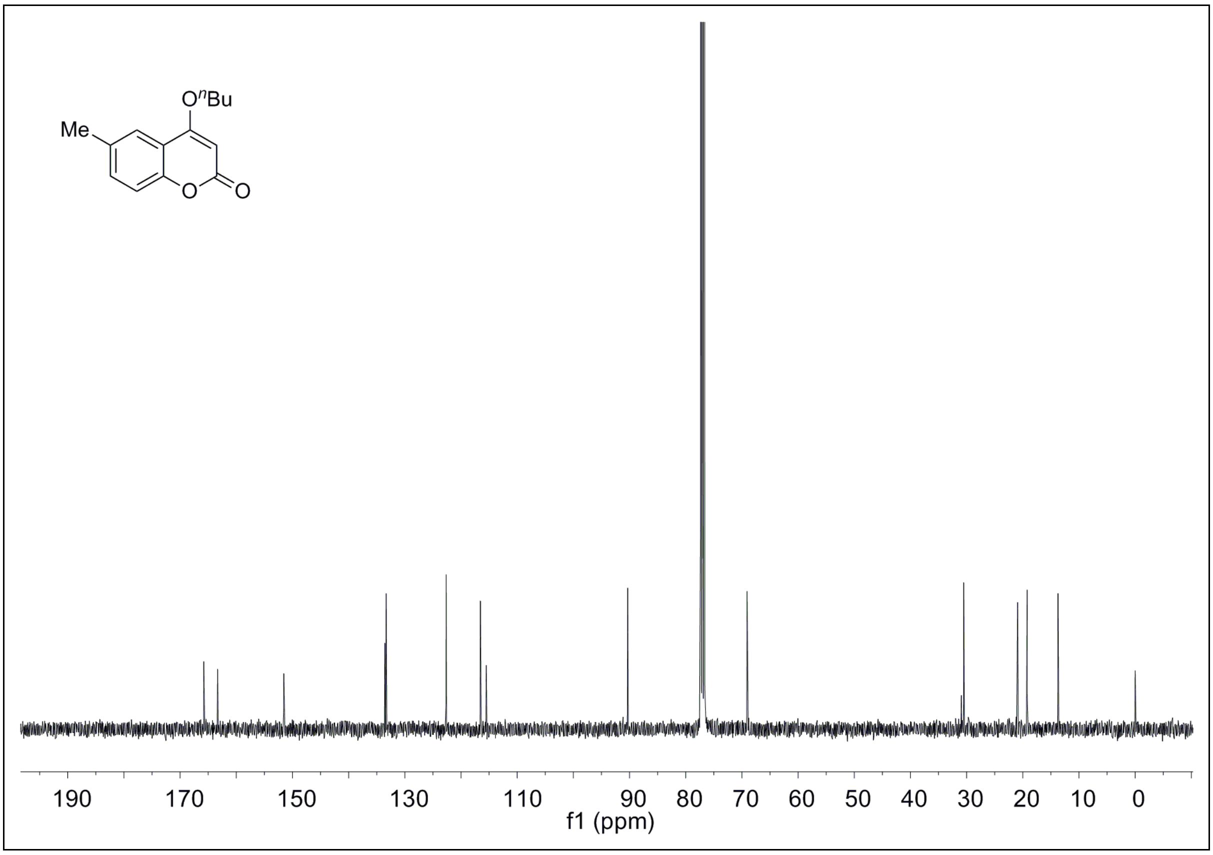
O^{n}Bu
Me
O
O
190
170
150
130
110
90
80
70
60
50
40
30
20
10
0
f1 (ppm)

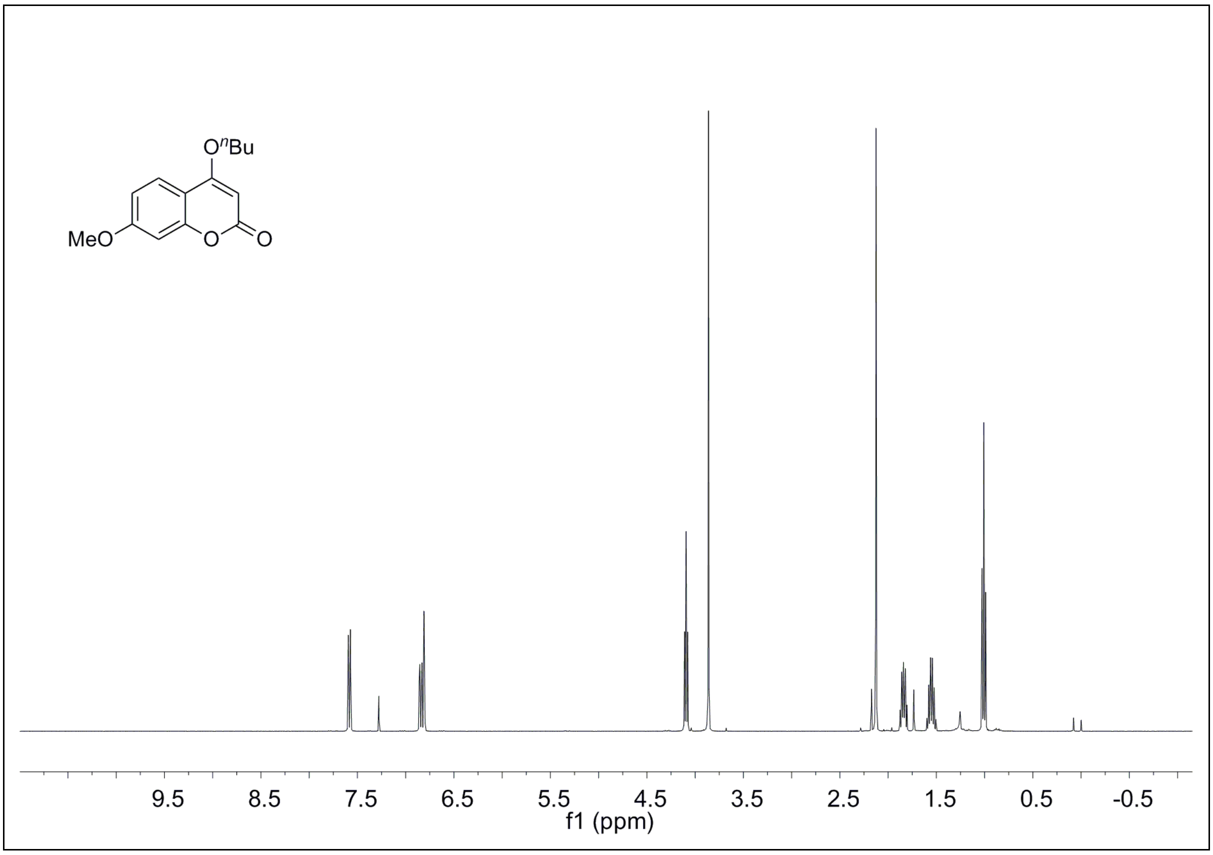
O^{n}Bu
MeO
O
O
9.5
8.5
7.5
6.5
5.5
4.5
3.5
2.5
1.5
0.5
-0.5
f1 (ppm)

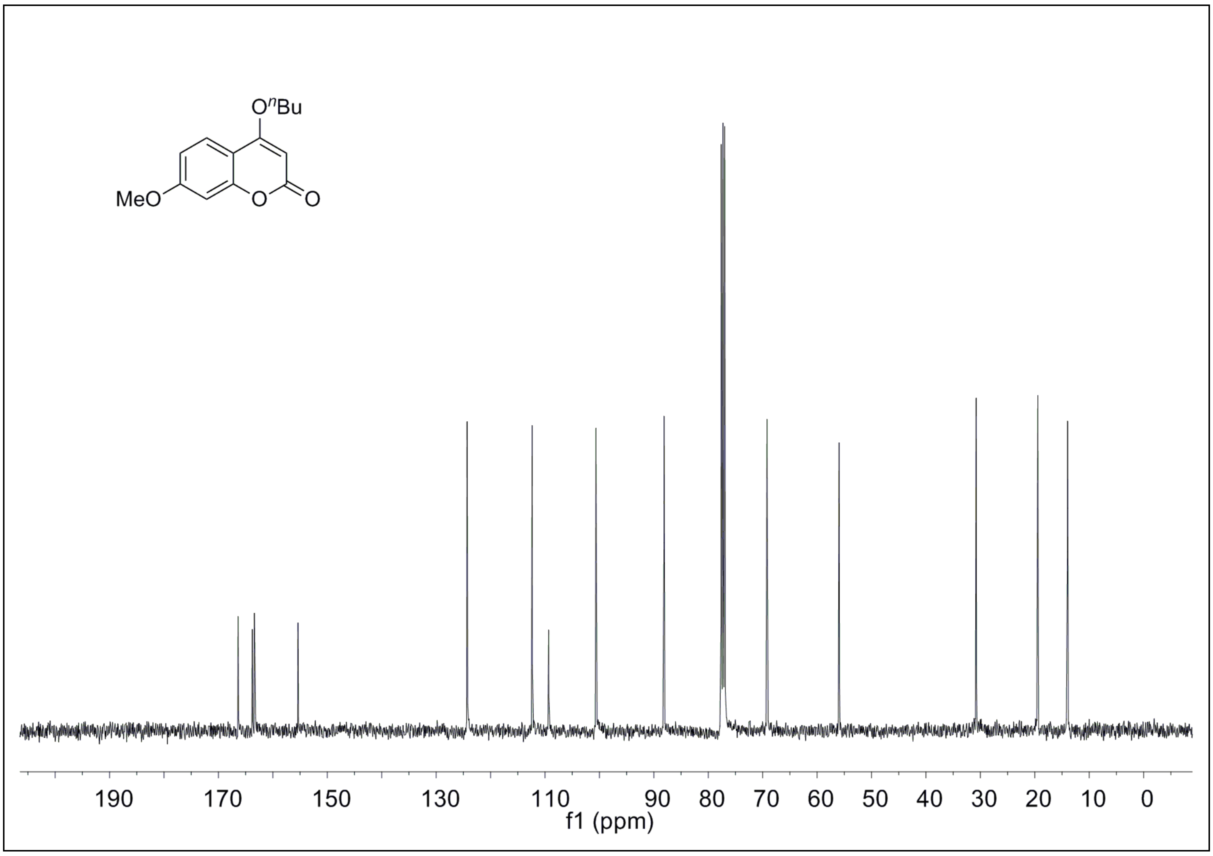
O^{n}Bu
MeO
O
O
190
170
150
130
110
90
80
70
60
50
40
30
20
10
0
f1 (ppm)

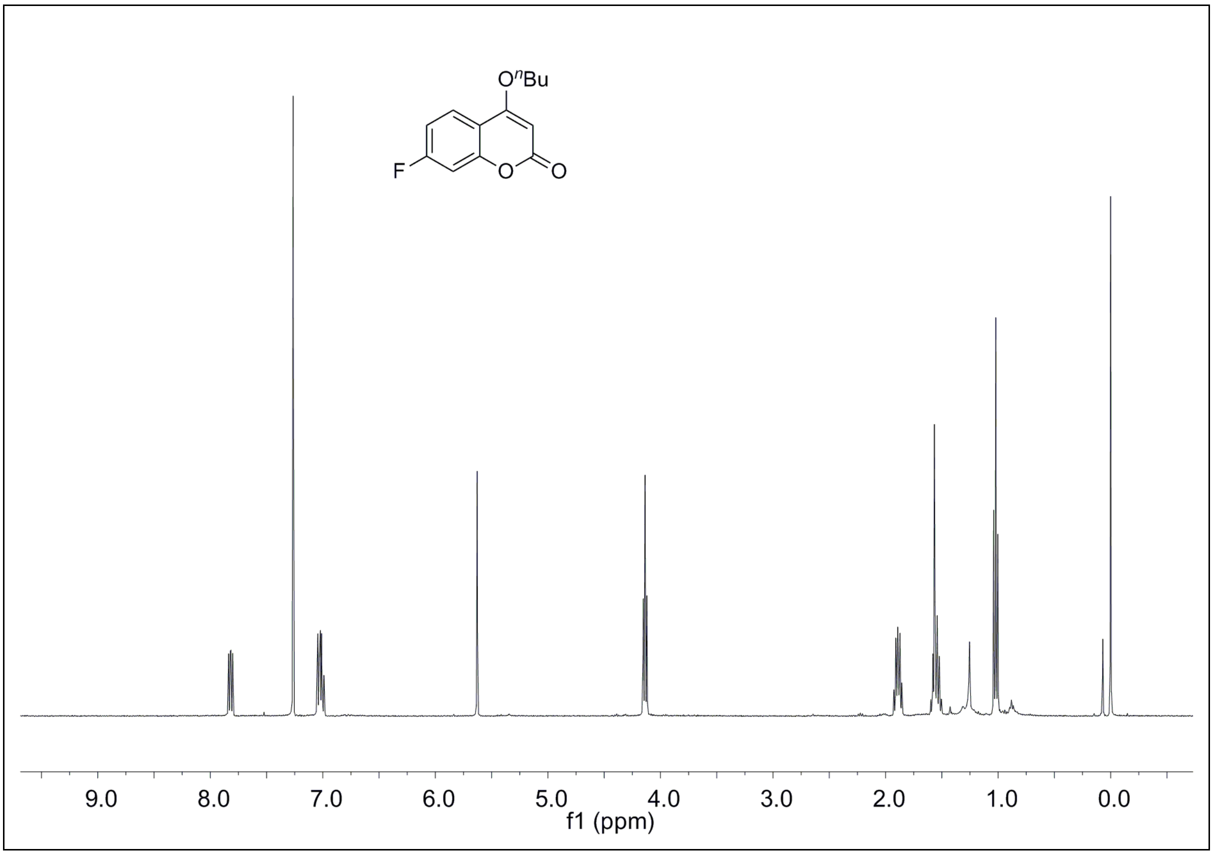
O^{n}Bu
F
O
O
9.0
8.0
7.0
6.0
5.0
4.0
3.0
2.0
1.0
0.0
f1 (ppm)

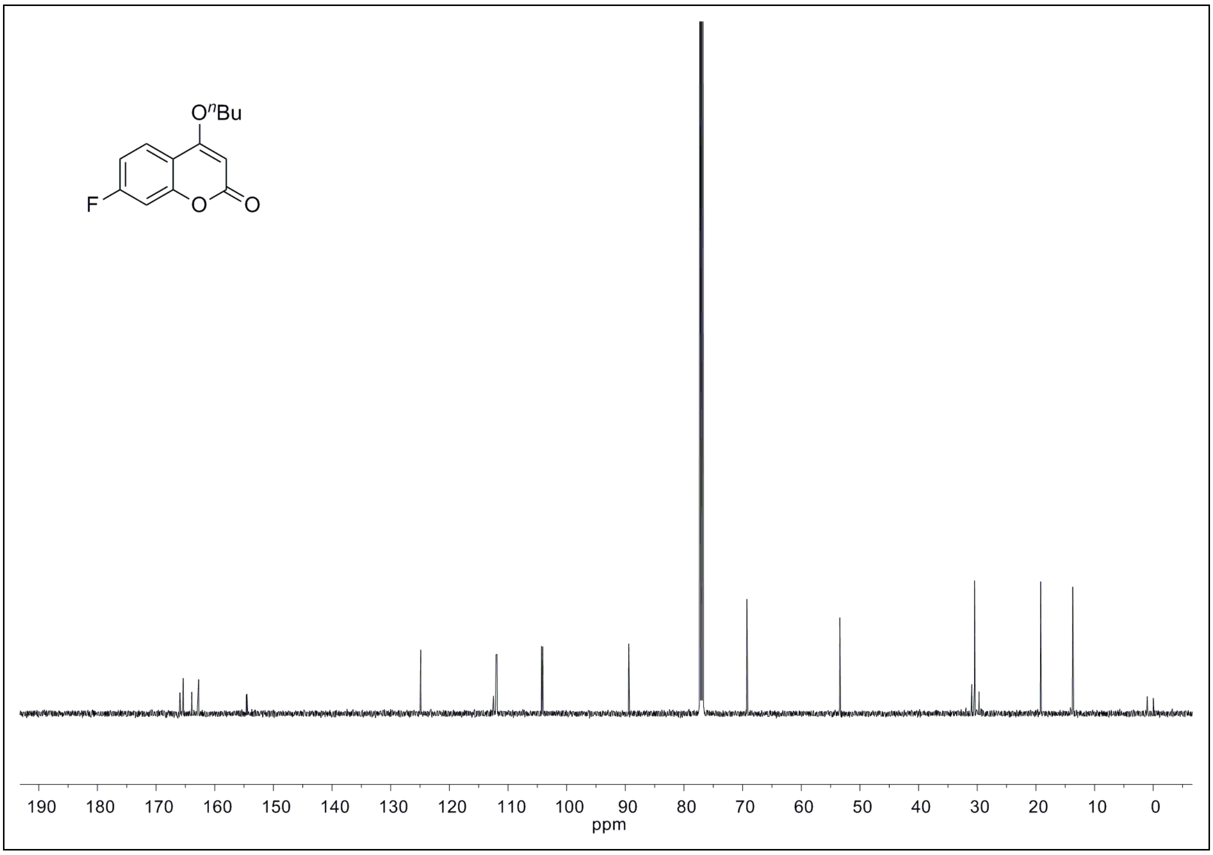
O^{n}Bu
F
O
O
190
180
170
160
150
140
130
120
110
100
90
80
70
60
50
40
30
20
10
0
ppm

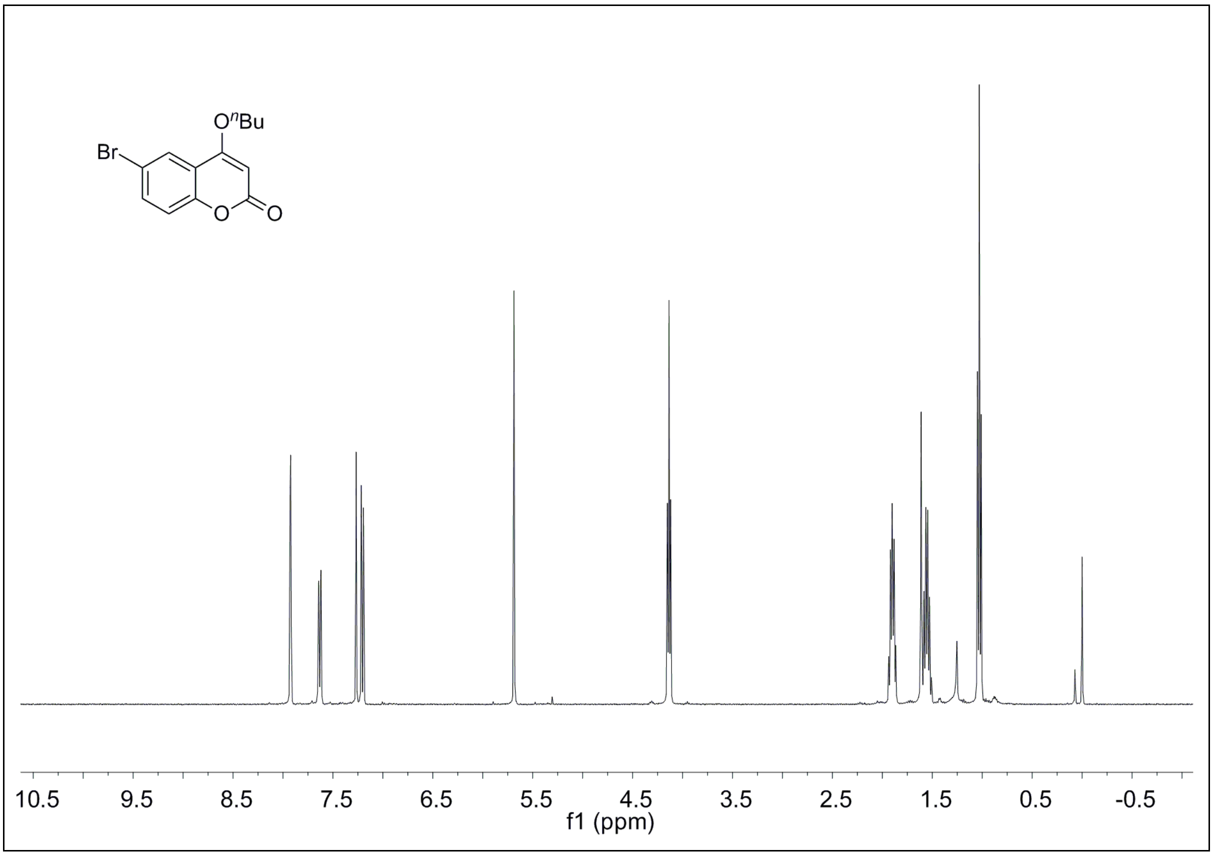
O^{n}Bu
Br
O
O
10.5 9.5 8.5 7.5 6.5 5.5 4.5 3.5 2.5 1.5 0.5 -0.5
f1 (ppm)

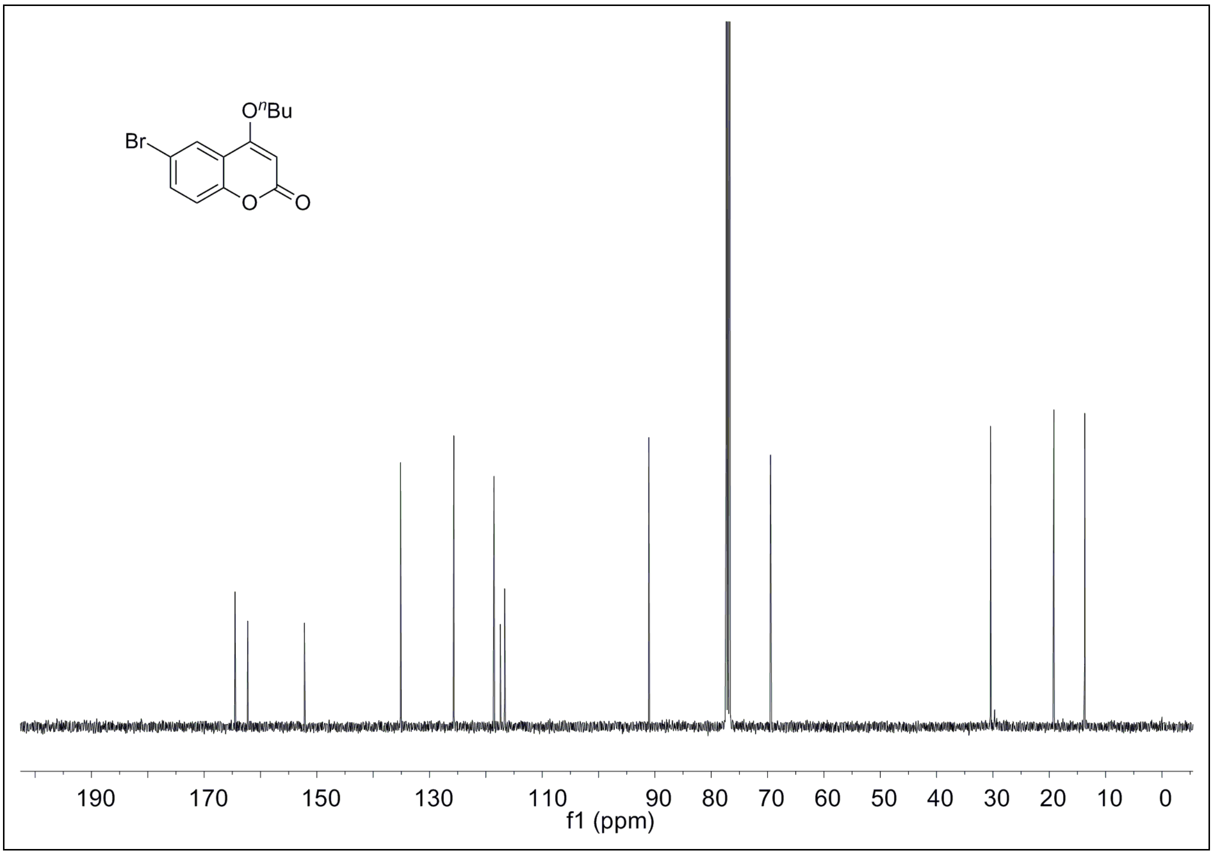
O^{n}Bu
Br
O
O
190 170 150 130 110 90 80 70 60 50 40 30 20 10 0
f1 (ppm)

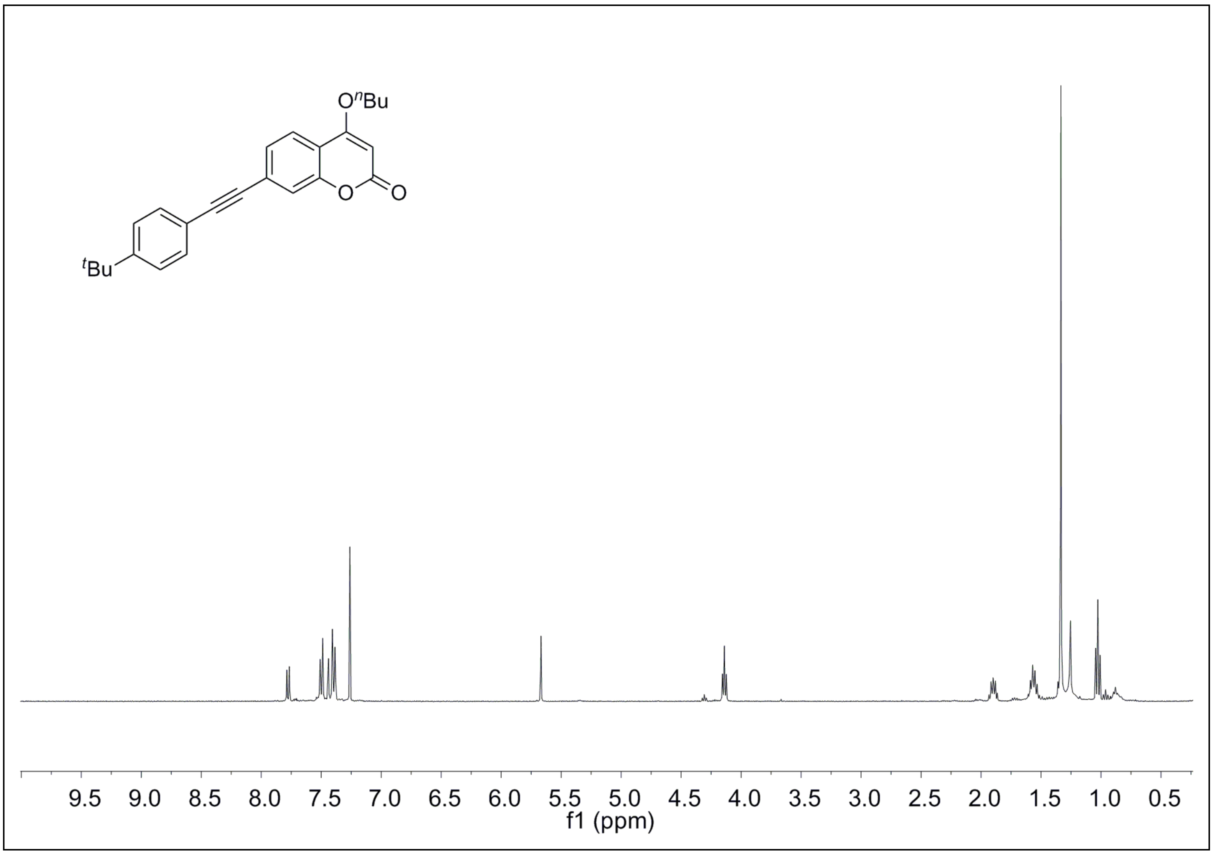

O^{n}Bu
O
O
^{t}Bu
9.5 9.0 8.5 8.0 7.5 7.0 6.5 6.0 5.5 5.0 4.5 4.0 3.5 3.0 2.5 2.0 1.5 1.0 0.5
f1 (ppm)

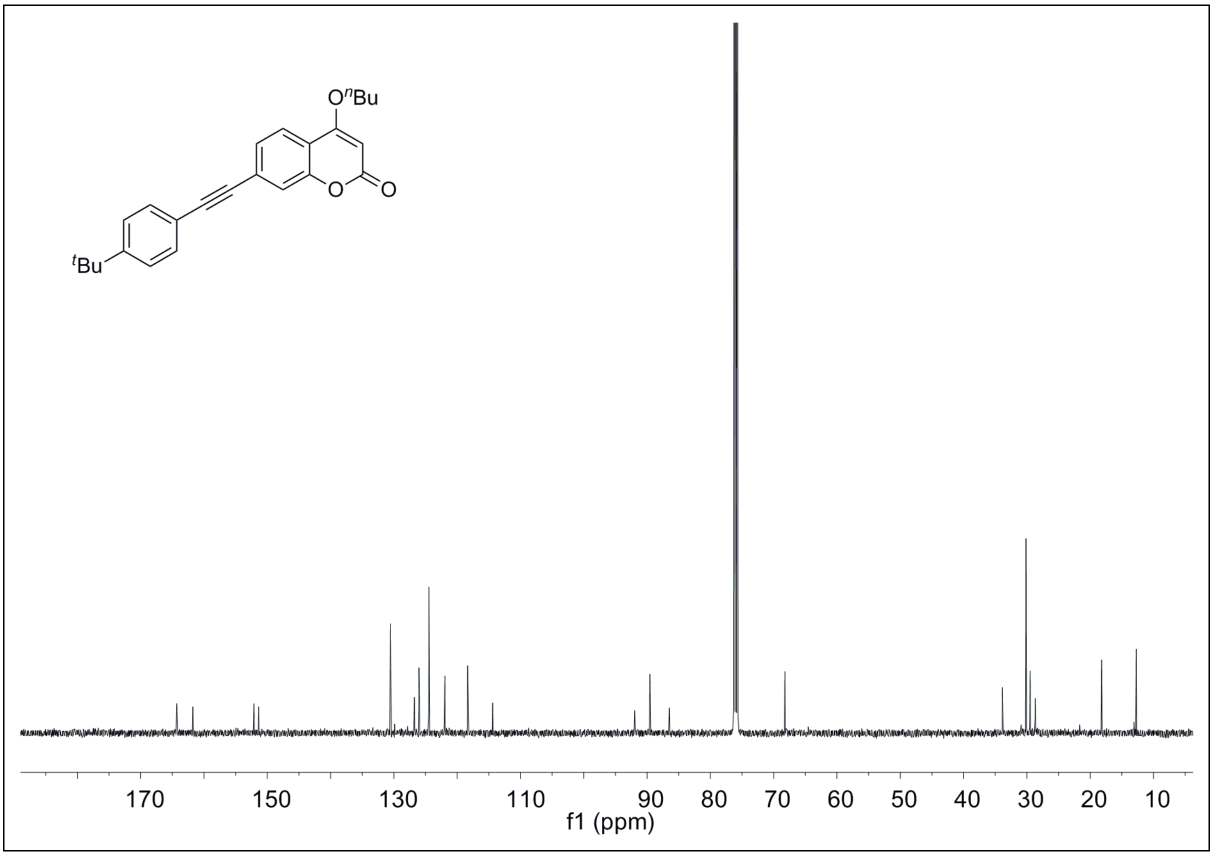
O^{n}Bu
O
O
^{t}Bu
170
150
130
110
90
80
70
60
50
40
30
20
10
f1 (ppm)

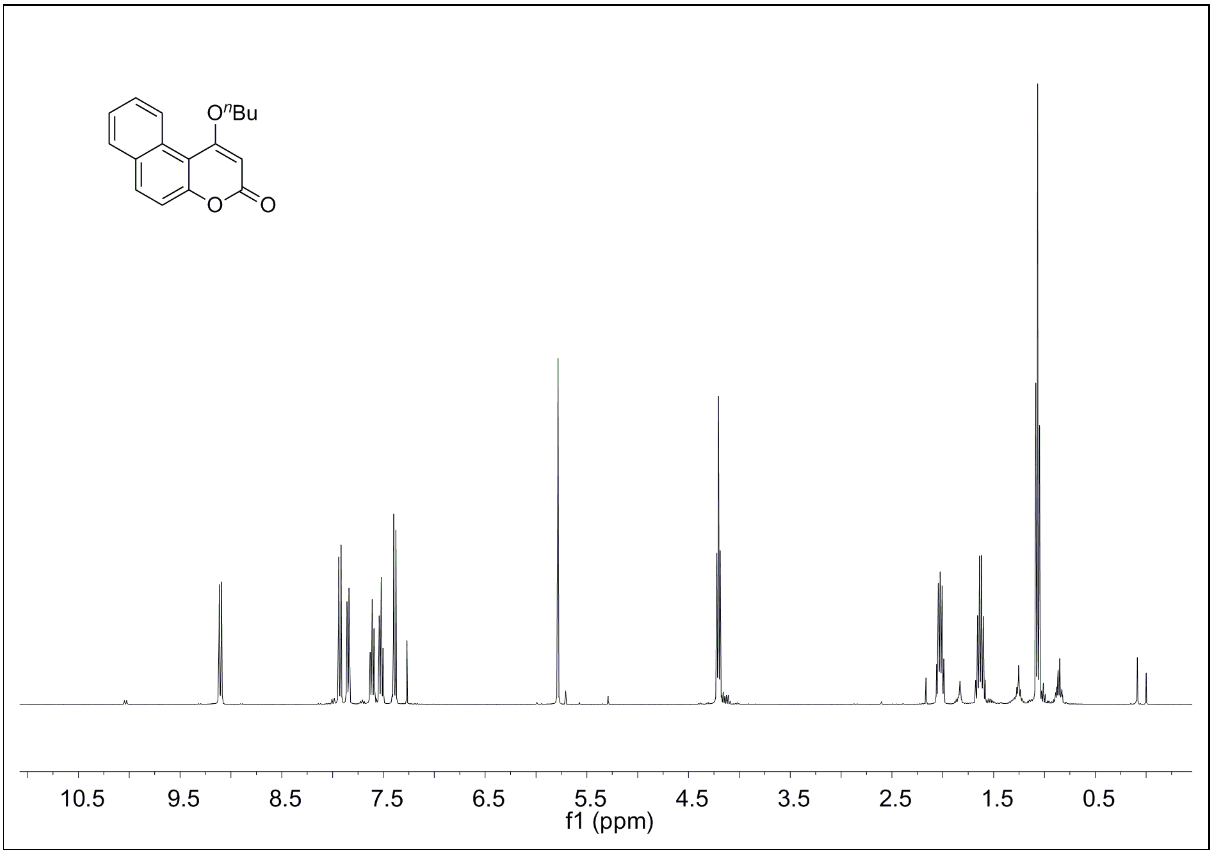
O^{n}Bu
O
O
10.5
9.5
8.5
7.5
6.5
5.5
4.5
3.5
2.5
1.5
0.5
f1 (ppm)

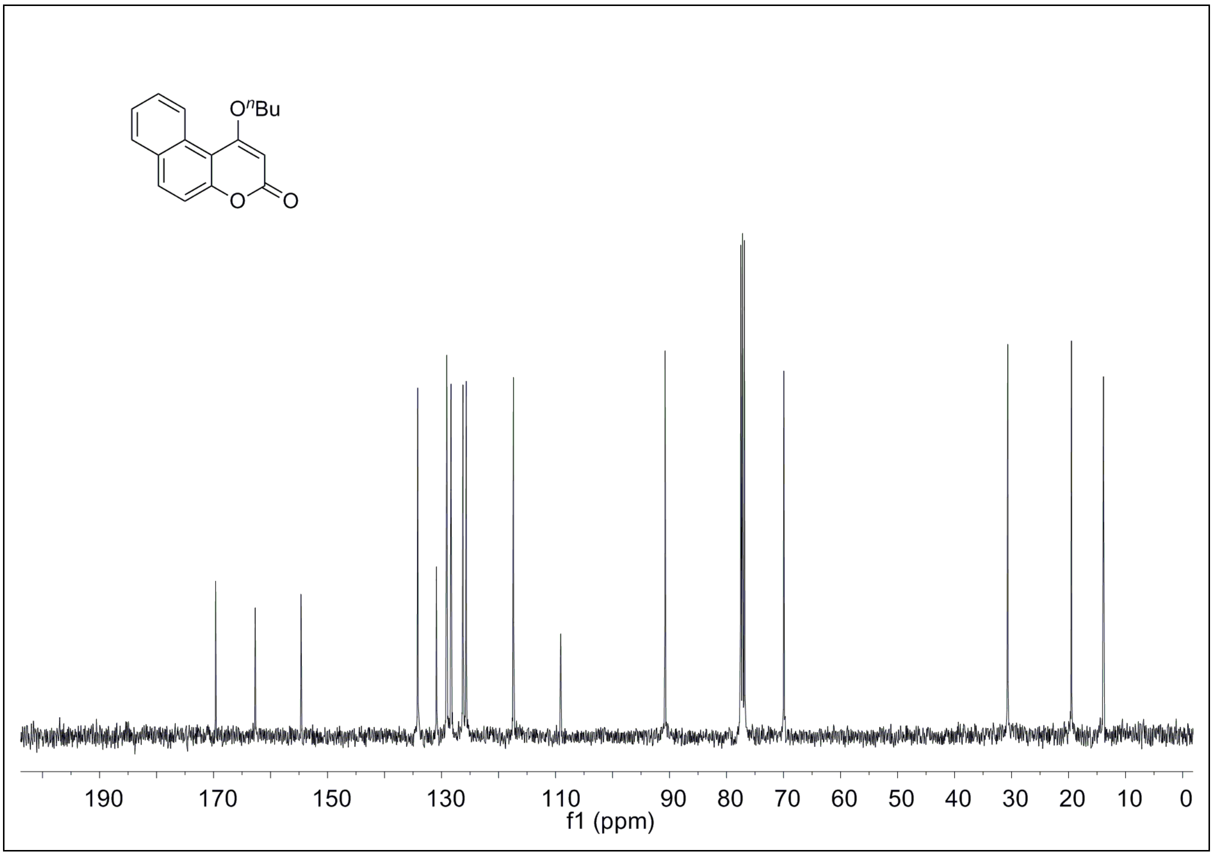
O^{n}Bu
O
O
190
170
150
130
110
90
80
70
60
50
40
30
20
10
0
f1 (ppm)

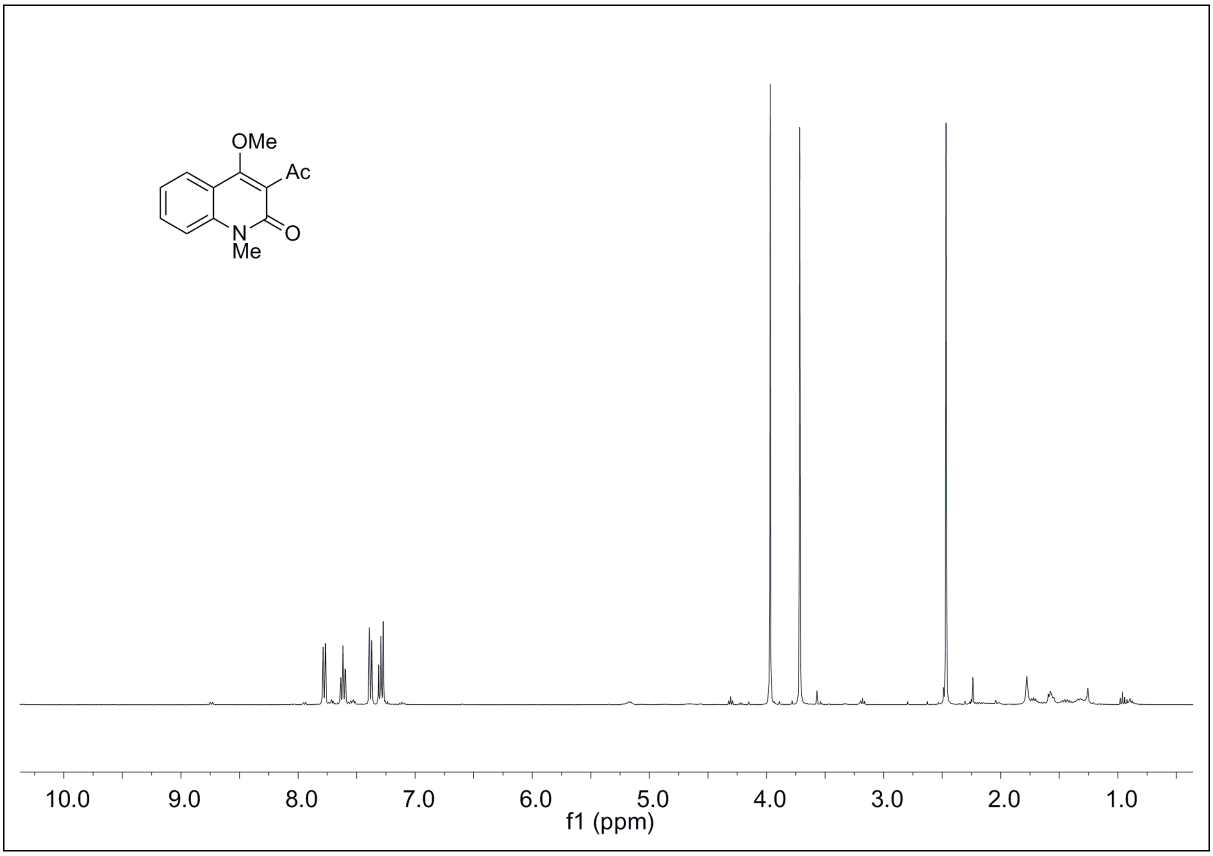
OMe
Ac
N
O
Me
10.0
9.0
8.0
7.0
6.0
5.0
4.0
3.0
2.0
1.0
f1 (ppm)

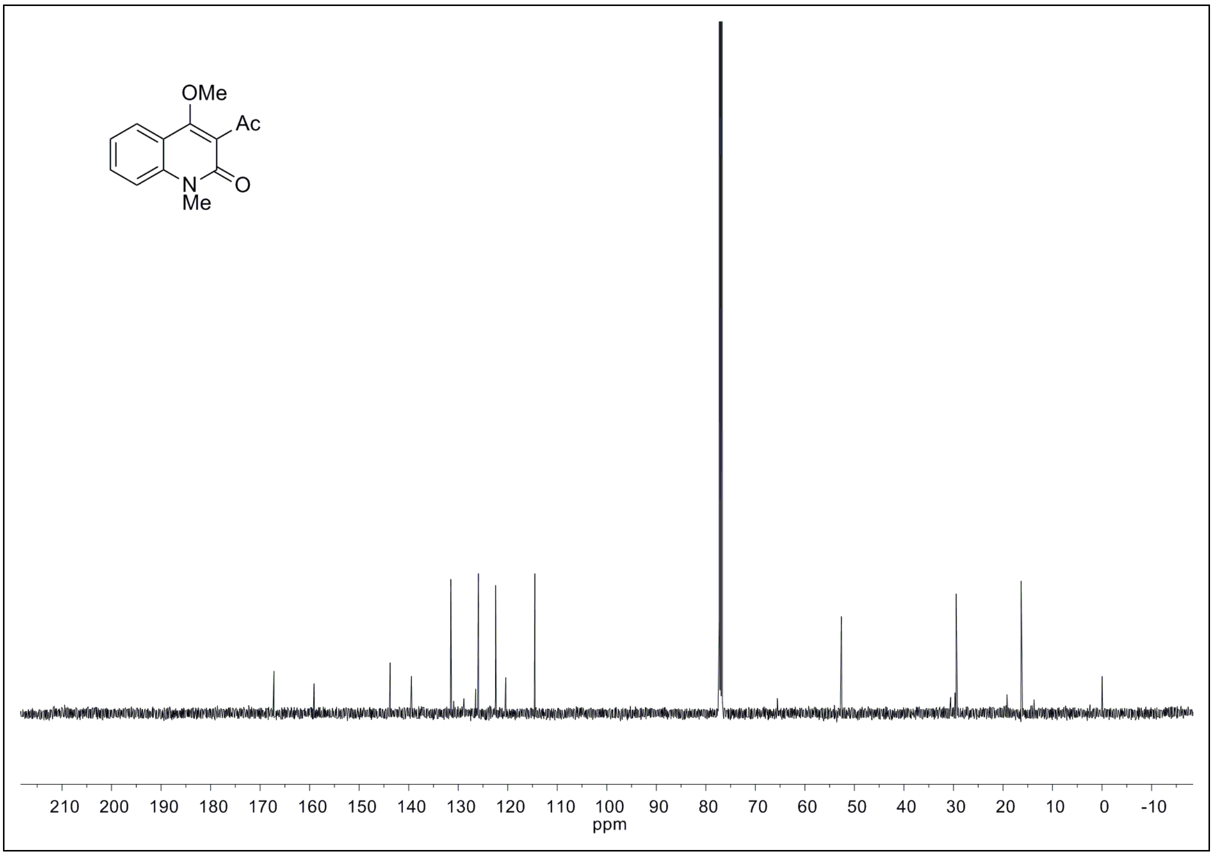
OMe
Ac
N
O
Me
210
200
190
180
170
160
150
140
130
120
110
100
90
80
70
60
50
40
30
20
10
0
-10
ppm

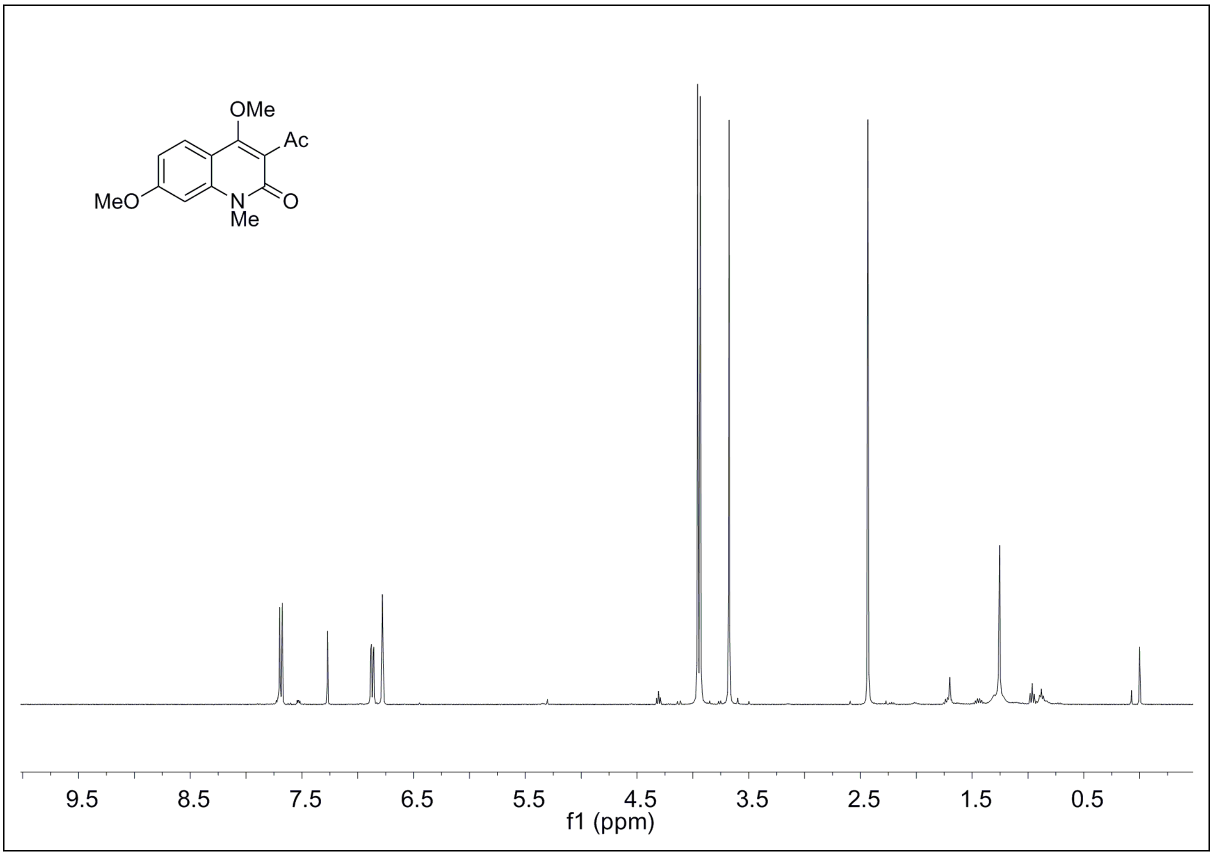
OMe
Ac
MeO
N
O
Me
9.5
8.5
7.5
6.5
5.5
4.5
3.5
2.5
1.5
0.5
f1 (ppm)

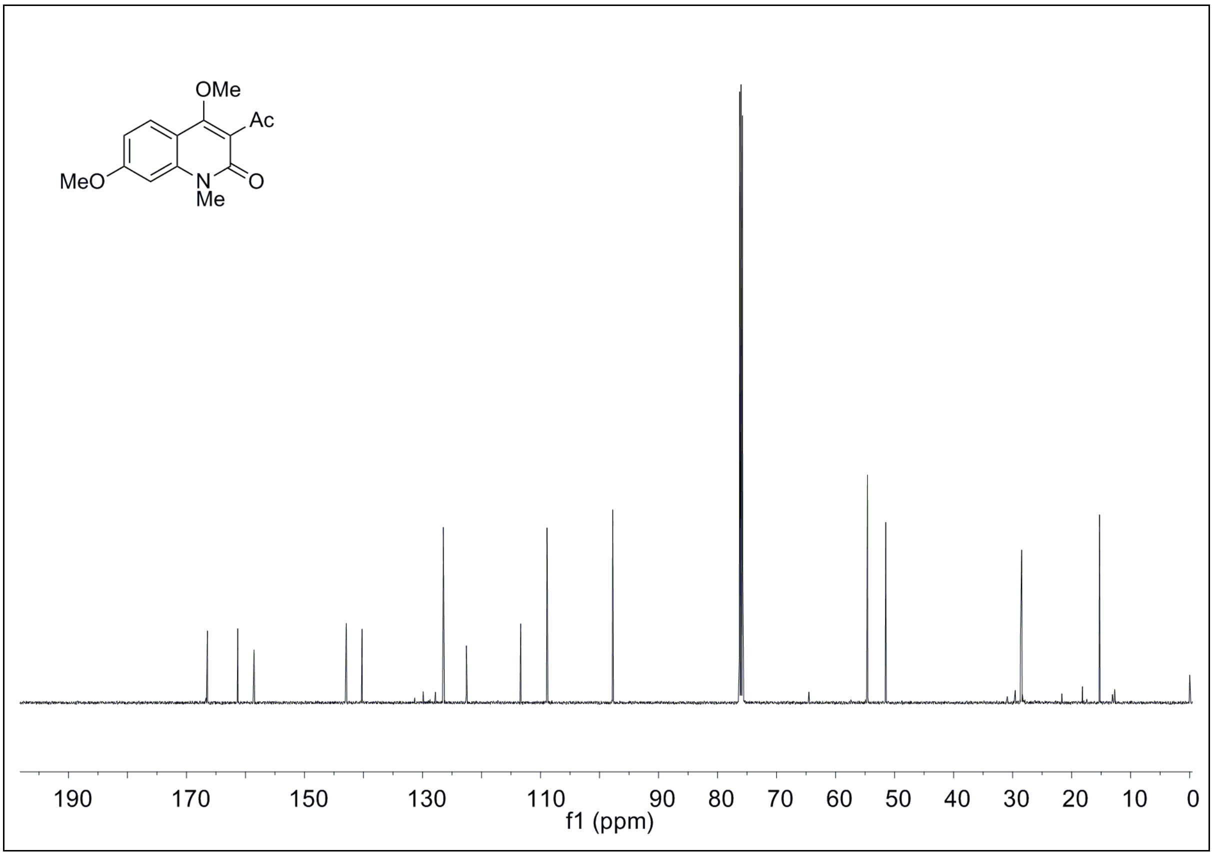
OMe
Ac
MeO
N
O
Me
190
170
150
130
110
90
80
70
60
50
40
30
20
10
0
f1 (ppm)

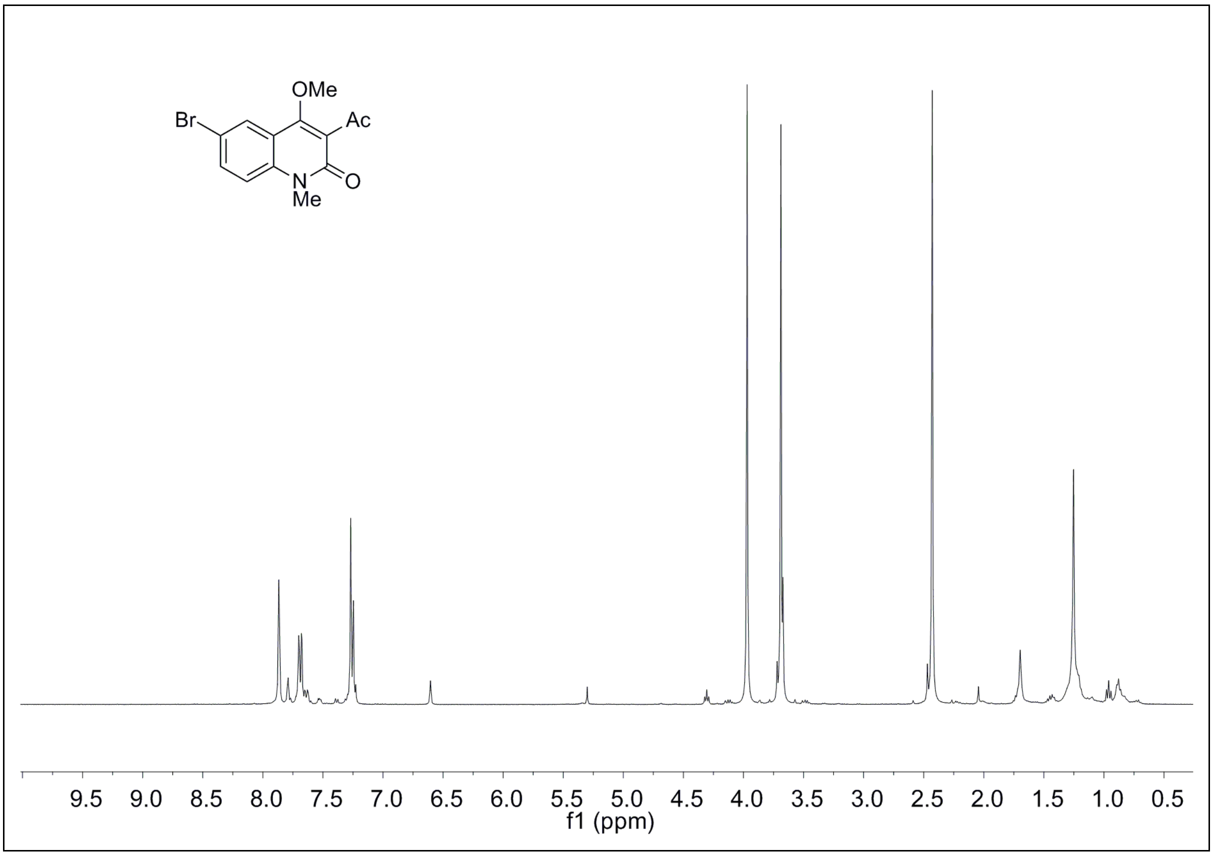
OMe
Br
Ac
N
O
Me
9.5 9.0 8.5 8.0 7.5 7.0 6.5 6.0 5.5 5.0 4.5 4.0 3.5 3.0 2.5 2.0 1.5 1.0 0.5
f1 (ppm)

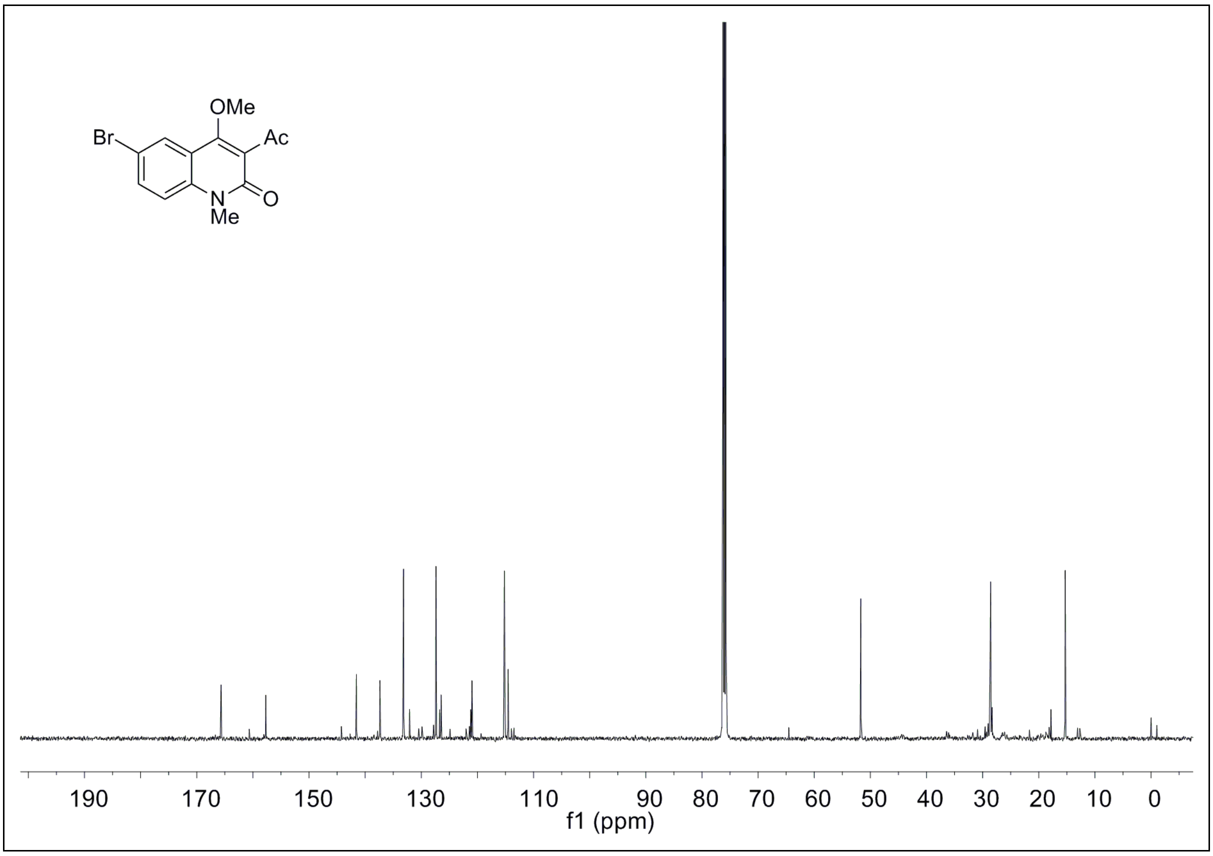
OMe
Br
Ac
N
O
Me
190 170 150 130 110 90 80 70 60 50 40 30 20 10 0
f1 (ppm)
